# Bioinspired organic materials for seamless neurohybrid interfaces: from material design to living electronics

**DOI:** 10.1039/d6mh00280c

**Published:** 2026-06-23

**Authors:** Nevena Stajković, Anna Manukânc, Matilde Trilli, Jan Steinkühler, Valeria Criscuolo, Francesca Santoro

**Affiliations:** a Institute of Biological Information Processing – Bioelectronics, IBI-3 Forschungszentrum Jülich 52428 Germany n.stajkovic@fz-juelich.de a.manukanc@fz-juelich.de f.santoro@fz-juelich.de; b Chair of Neuroelectronics, Faculty of Electrical Engineering and IT, RWTH Aachen University 52074 Aachen Germany criscuolo@nei.rwth-aachen.de; c Bio-Inspired Computation, Institute of Electrical and Information Engineering, Kiel University Kiel Germany jst@tf.uni-kiel.de; d Kiel Nano, Surface and Interface Science KiNSIS, Kiel University Kiel Germany

## Abstract

The brain architecture is multi-layered, with billions of neurons organized into intricate neural networks that communicate *via* synapses. Since synapse dysfunction is a main hallmark of neurological disorders, understanding the mechanisms underlying their failure is necessary for designing novel treatments. Neuroelectronic devices are powerful tools for recording and stimulating brain activity. Therefore, they can be used to understand and potentially treat neurological disorders. Achieving this requires the seamless integration of neurohybrid interfaces into neural tissue, enabling bidirectional communication. This focus article highlights the roles of organic mixed ionic-electronic conductors, advanced polymer chemistry, and soft materials with tunable properties in enabling seamless integration. Drawing inspiration from the dynamic structure of neurons, we cover materials that mimic neuron structure and function. We explore novel approaches, including *in vivo* polymerization and synthetic biology, that hold promise for realizing living neuroelectronics and advancing the frontiers of neuroscience, bioengineering, and clinical medicine.

## Abstract 2

Inspired by neurons, soft organic mixed ionic-electronic conductors, synthetic biology, and *in situ* polymerization open new avenues toward living neuroelectronics. Created in BioRender and Procreate®. Santoro, F. (2026) https:/BioRender.com/t87jwqy.

## Graphical abstract

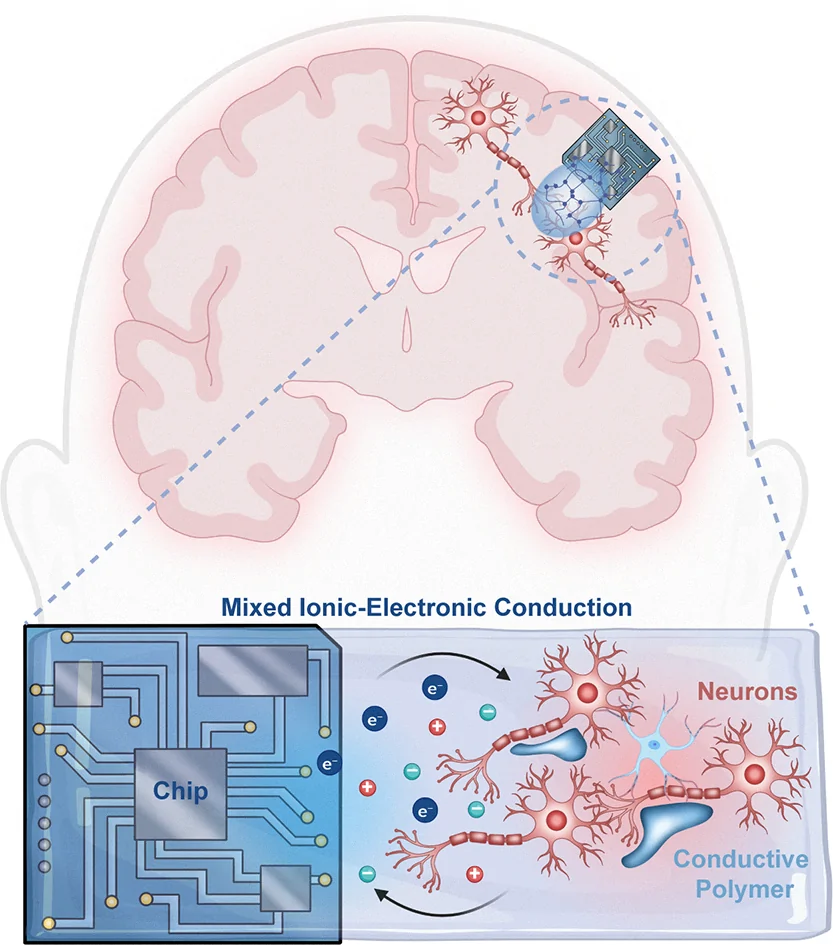

## 1. Introduction

The nervous system coordinates body functions and regulates autonomic processes essential for survival, such as cardiac rhythm, respiration, and metabolism.^1^ Beyond these fundamental roles, it supports a wide range of complex sensorimotor and cognitive functions.^1^ Central to these high-order processes is the brain, a network composed of approximately 86 billion neurons that shapes behavior, enables learning and memory formation, supports problem-solving and artistic expression, and controls precise motor skills required for activities such as dancing or running.^2^ Through these functions, the brain ultimately shapes perception and individual identity. Despite extensive research, the precise molecular mechanisms underlying brain function under both physiological and pathological conditions remain incompletely understood. Gaining a deeper understanding of neural physiology and communication, particularly at the synaptic level, is therefore critical. Given that synaptic dysfunction is a hallmark of numerous neurodegenerative and psychiatric disorders,^3–5^ understanding these mechanisms would be a breakthrough for new diagnostic and therapeutic tools.^6^

**Fig. 1:**
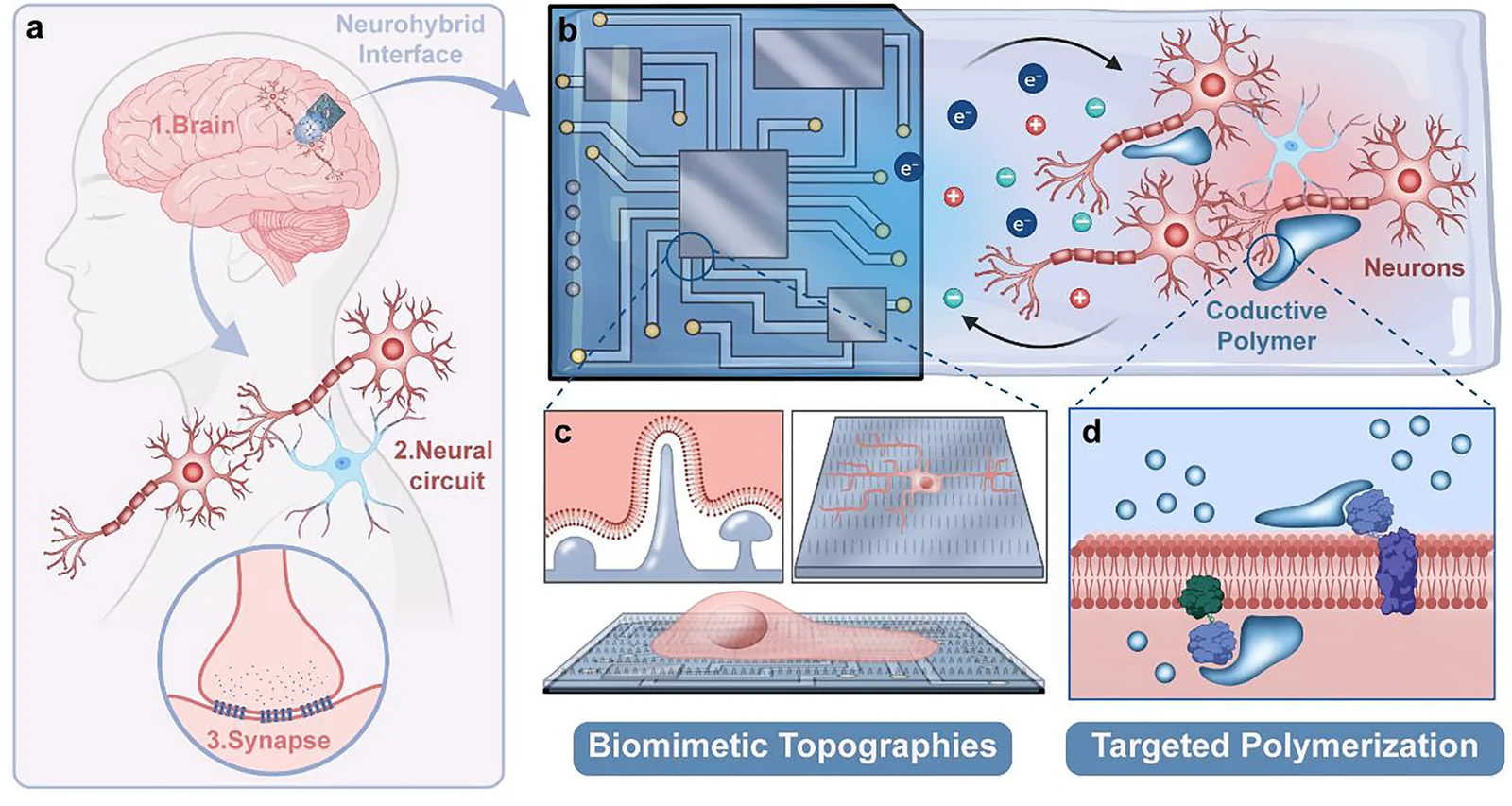
Strategies for seamless neurohybrid interfaces and living neuroelectronics. (a) Brain organization spans multiple scales, from macroscopic brain regions to neural circuits and synapses. *In vivo* electronics offers an opportunity to study and repair neural functions but requires adaptive biomimetic interfaces that integrate with neural tissue at cellular and subcellular levels. (b) Organic mixed ionic-electronic conductors and conductive polymers are widely explored for neurohybrid interfaces and promising candidates for living electronics due to their biocompatibility, mixed ionic-electronic conduction, and tunability. (c) Biomimetic topographies are indispensable for living neuroelectronics, as they modulate neuronal adhesion, growth, and network formation while enhancing cell–chip coupling. (d) Targeted *in situ* polymerization enables localized polymer deposition at a defined cellular compartment, providing a key route toward fully integrated neurohybrid devices. Schemes were created in BioRender. Santoro, F. (2026) https://BioRender.com/dsqbqhq with an additional object generated in Procreate®. Panel (d) was inspired by ref. 7 and 8.

Neuroelectronic technologies capable of interacting with the brain provide powerful tools for recording and modulating neural activity, thereby supporting investigations into unresolved mechanisms underlying synaptic plasticity.^9^ Advances in neuroelectronics have highlighted the pivotal role of electronic devices as neural interfaces and neuroprosthetics in understanding, diagnosing, and treating neurological diseases by establishing intimate contact between artificial and biological domains. In this context, the controlled and precise implementation of interfaces is crucial for repairing injured tissue or designing novel devices for brain stimulation and recording. Recently, the concept of neurohybrid^10,11^ has emerged to describe systems that bridge biological neurons with artificial devices, in which established physical connections enable a unidirectional or bidirectional information exchange (Fig. 1). In addition, the new field of neuromorphic engineering has shown great promise in developing electronic devices that can emulate neural computation capabilities, featuring low power consumption, plasticity, and learning. The concept of neuromorphic can be further expanded, drawing from the ancient Greek *morphē* for “shape”, to include devices that mimic not only the information processing ability of the brain, but also its key morphological features.

Both *in vitro* and *in vivo* platforms can be engineered to realize neuroinspired integrated systems. However, the brain's complex architecture, encompassing a multiscale hierarchy from macroscopic structures to cellular and molecular levels (Fig. 1), imposes several requirements. Consequently, the choice of materials' chemistry and composition, topography, spatial arrangement, and electrical properties is of paramount importance. In the light of neuroinspired systems, organic chemistry and polymer science offer virtually infinite possibilities for mimicking physiological pathways. A particularly promising frontier is the recent advance in the polymerization of conductive materials directly within living tissues (Fig. 1). This approach paves the way for realizing living electronics, in which electronic devices are fully integrated with living tissue to function as a single system. In this focus article, we discuss how recent materials advancements, from chemical and structural perspectives, are bridging the gap between neuroscience and technology by actively mimicking neural features and integrating and exploiting biological functions.

## 2. Signal transmission in the nervous system: from the brain to the synapse

**Fig. 2:**
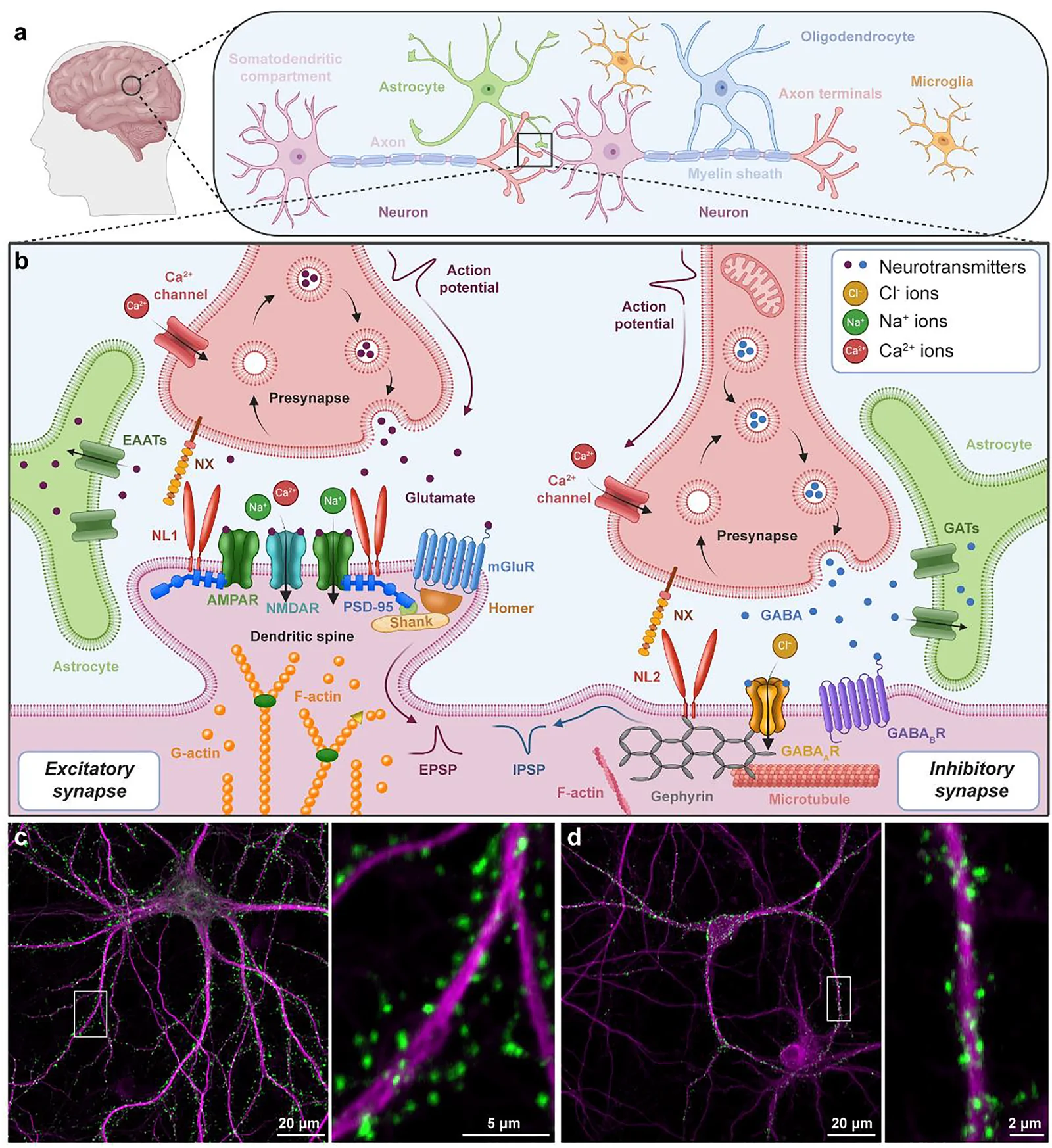
Organization of the brain and synapses. (a) Schematic representation of the brain organization at the cellular level, including neurons, oligodendrocytes, astrocytes, and microglia. Major neuronal compartments are indicated: synapses (boxed region), soma and dendrites, myelinated axons, and axon terminals. (b) Schematic depiction of the molecular organization of excitatory (left) and inhibitory synapses (right), highlighting key ion channels and receptors (*e.g.*, NMDAR, AMPAR, GABA_A/b_R), cell adhesion molecules, and scaffolding proteins. Abbreviations: Nl, neuroligin; Nx, neurexin; AMPAR, α-amino-3-hydroxy-5-methyl-4-isoxazolepropionic acid receptors (AMPAR); NMDAR, *N*-methyl-d-aspartate receptors; mGluR, metabotropic glutamate receptor; EAATs, excitatory amino acid transporters; GAT, GABA transporter; F-actin, filamentous actin; EPSP, excitatory postsynaptic potential; IPSP, inhibitory postsynaptic potential. Schemes in a and b are created in BioRender. Santoro, F. (2026) https://BioRender.com/hzs2xe4. Some objects were generated in Procreate® and imported into BioRender. Inspired by ref. 15 and 16. (c) and (d) Confocal images of primary neurons immunostained for the dendritic marker, MAP2 (microtubule-associated protein-2), and markers of excitatory (PSD-95) and inhibitory (GABA_A1_R) post-synapses. The synaptic staining shown here does not represent new research data, but serves as an illustrative example reproduced in our laboratory using established immunostaining approaches. Primary antibodies included an extracellular GABA-A receptor α1 subunit antibody (224 211, Synaptic Systems), MAP2 (ab5392, Abcam), and PSD95/MAGUK scaffold protein antibody (NeuroMab clone K28/43). All secondary antibodies were obtained from Thermo Fisher Scientific. Boxed regions in the left panels indicate synapses shown at higher magnification in the right panels.

The brain is the body's command center, receiving and integrating signals and generating context-appropriate outputs that are transmitted to other neurons or effector cells through electrical (action potentials) and chemical (neurotransmitters) signals.^12^ At the cellular level, these capabilities arise from the activity of neurons, highly specialized, excitable cells organized into circuits to receive, compute, encode, and relay information (Fig. 2a).^12^ Information processing and transmission within neural circuits occur at chemical synapses, which are dynamic structural and functional connections between neurons (Fig. 2a and b).^13^ With an estimated 100–1000 trillion synapses in the human brain,^14^ these activity-dependent structures underlie neural communication, learning, and memory formation.^6^

At the chemical synapses, presynaptic axon terminals release neurotransmitters that bind to receptors on the postsynaptic neuron, typically located on dendritic spines or shafts,^17^ and occasionally somata or axons (Fig. 2b–d).^5^ Neurotransmitter binding induces conformational changes in receptors, enabling ion flux across the membrane and resulting in membrane hyperpolarization or depolarization (Fig. 2b).^12^ Individual neurons integrate excitatory and inhibitory inputs from thousands of synapses and, upon reaching the threshold, generate action potentials at the axon initial segment.^18^ These electrical signals then propagate along the axon to the terminals, where they trigger neurotransmitter release, thereby relaying information through the neural circuit.^6^

In addition to chemical synapses, neurons can communicate directly *via* electrical synapses.^19^ These connect the cytoplasm of neighboring neurons *via* clusters of intercellular channels (connexins) that form a gap junction.^20^ Such an organization enables rapid, bidirectional transmission of electrical signals and the exchange of small molecules between neurons.^19,20^ Moreover, a recent study demonstrated that neurons can communicate directly *via* non-synaptic nanotubular bridges formed between dendrites to exchange small molecules, ions, and even pathogenic proteins.^21^

In addition to neurons, glial cells account for approximately half of the brain's volume, including oligodendrocytes, astrocytes, microglia, and oligodendrocyte precursor cells (Fig. 2a).^22^ Neuron–glia interactions are crucial for the optimal nervous system development and function, as well as the maintenance of homeostasis.^23^ Astrocytes establish intimate contact with synapses (Fig. 2b) and play an active role in synaptogenesis, dendritic spine maturation, modulation of synaptic plasticity, synapse refinement, and axon pruning.^22,23^ Microglia, the brain's immune cells, also contribute to synapse formation and refinement.^23^ Oligodendrocytes form the myelin sheath (Fig. 2a) in the central nervous system (CNS), enabling the rapid propagation of action potentials within neuronal networks by reducing membrane capacitance.^22^

### 2.1 Diversity of synaptic connections in neural circuits

Based on a neurotransmitter released from the presynaptic terminal, synapses are classified as excitatory, inhibitory, or modulatory.^24^ Excitatory synapses, predominantly glutamatergic in the adult CNS, increase the likelihood of action potential generation in postsynaptic neurons (Fig. 2b).^24^ Inhibitory synapses, mainly γ-aminobutyric acid (GABA)–ergic in the adult CNS, account for 5–20% of all brain synapses^5^ and reduce firing probability (Fig. 2b).^24^ Modulatory synapses release neurotransmitters such as dopamine or serotonin to regulate broader network activities.^24^ In addition, co-transmission synapses contribute to complex signaling by releasing multiple neurotransmitters from a single synapse.^24^

Distinct synapse types exhibit specialized proteome compositions, comprising cell adhesion molecules (CAMs), scaffolding proteins, and ion channels that assemble into unique nanoscale architectures, thereby determining synaptic identity and function (Fig. 2b–d).^24^ Transmembrane CAMs, including presynaptic neurexins and postsynaptic neuroligins, form transsynaptic connections that promote the formation of functional synapses.^4,25,26^ Specific neuroligins (Nl) isoforms interact with scaffolding proteins, post-synaptic density protein 95 (PSD95), and gephyrin, at excitatory or inhibitory synapses, respectively (Fig. 2b).^4^ In addition to neuroligin, PSD95 interacts with glutamate receptors, playing a crucial role in stabilizing excitatory synapses and regulating synaptic plasticity (Fig. 2b).^4^ The α-amino-3-hydroxy-5-methyl-4-isoxazolepropionic acid receptors (AMPAR), *N*-methyl-d-aspartate receptors (NMDAR), and metabotropic glutamate receptors (mGluR) are enriched in the postsynaptic density of the dendritic spines, along with ∼1000 other proteins that form a dense molecular network (Fig. 2b).^17^ These glutamate receptors also interact with scaffolding proteins such as Shank and Homer (Fig. 2b).^17^ At inhibitory post-synaptic sites, ionotropic chloride channels—primarily GABA type A receptors (GABA_A_R) and glycine receptors—are clustered on dendritic shafts (Fig. 2b and d), in the peri-somatic region, or at the axon initial segment to control output of the postsynaptic neurons.^5,17^ Notably, in approximately 30% of cases,^27^ inhibitory synapses are transiently established on dendritic spines, underscoring the complexity of neuronal organization.^28^

Presynaptic terminals are enriched in voltage-gated calcium channels (Ca_V_) that open in response to incoming action potentials (Fig. 2b).^6^ The resulting elevation in intracellular Ca^2+^ concentration within the active zone triggers the exocytosis of synaptic vesicles with neurotransmitters.^6,13,29^ In addition to neurexins,^25^ the key presynaptic membrane proteins include syntaxin and synaptosomal-associated protein 25 (SNAP25).^29^ Together with vesicular synaptotagmin, these proteins form the SNARE (SNAP soluble NSF attachment protein receptor) complex, which mediates synaptic vesicle fusion and neurotransmitter release.^29^ Other prominent presynaptic proteins include synaptophysin and synapsin.^29^ Presynaptic diversity is particularly prominent among GABAergic interneurons, which establish synapses on excitatory neurons or other interneurons to regulate neuronal output and fine-tune information integration within neural circuits.^5,13^

Short and long branches of filamentous actin (F-actin) constitute key components of the dendritic spine cytoskeleton (Fig. 2b), where they define spine morphology.^30^ Accordingly, remodeling of the actin cytoskeleton network plays a major role in structural synaptic plasticity.^13,30^ At presynaptic sites, actin is organized into heterogeneous structures, including actin nanomesh, rails, and corrals, that facilitate clustering of presynaptic components and vesicle recycling.^31^

### 2.2 Synaptic plasticity

Synaptic plasticity refers to structural and functional changes of synapses that occur in response to neural activity, resulting in modifications of synaptic strength and efficiency.^13,23^ Structural plasticity involves synapse remodeling, characterized by alterations in the number, shape, and size of synapses. At the molecular level, these morphological changes are mirrored by alterations in the composition and abundance of synaptic proteins, such as AMPAR and NMDAR, as well as by remodeling of the actin cytoskeleton.^5,30^ Functional plasticity, in contrast, involves activity-dependent changes in the neurotransmitter release.^23^

In mammals, synaptic plasticity can be classified as short- or long-term, depending on the duration of the change. Short-term plasticity is characterized by transient changes in the effectiveness of neurotransmitter release lasting from milliseconds to minutes. Long-term plasticity, in contrast, involves structural and functional remodeling at pre- and postsynaptic sites, resulting in long-term alterations in synaptic strength. Structural long-term potentiation (LTP) and long-term depression (LTD) are associated with enlargement and reduction of spine head size, respectively. LTP is widely considered a primary mechanism underlying neural circuit reorganization during learning and memory formation.^23,30^ At the molecular level, the induction of LTP and LTD drives the insertion and removal of AMPAR from the post-synaptic membrane, respectively, making AMPAR modulation crucial for rapid changes in synaptic strength.^32^

To prevent destabilization of network activity arising from these synaptic modifications, homeostatic plasticity maintains overall neuronal network stability by coordinating plasticity across cellular and subcellular compartments.^23^ Homeostatic plasticity encompasses mechanisms that are regulated across neurons and glial cells, compensating for perturbations in neuronal activity or synaptic transmission by dynamically adjusting synaptic strength and cellular properties.^33^ Despite widespread recognition of the central role of synapses in information processing and encoding, a detailed molecular dissection of the underlying mechanisms remains incomplete.^34^ Identifying the molecular pathways that govern synaptic formation, signaling, and plasticity is therefore essential to advancing our understanding of both physiological and pathological brain function.^6^

## 3. From physiology to engineering neuroinspired materials

Neuroelectronic devices, such as multi-electrode arrays (MEAs, also known as micro-electrode arrays), have transformed both *in vitro* and *in vivo* electrophysiology over the past decades, enabling the long-term monitoring of neuronal network activity.^35^ While the gold-standard patch-clamp technique can measure ionic currents from single cells and detect intracellular subthreshold signals, it does so at the expense of neuronal viability and experimental throughput.^36^ In contrast, MEAs offer a high-throughput, minimally invasive alternative that holds promise for developing neurohybrid constructs, especially given the broad spectrum of available fabrication technologies.^37,38^ This design flexibility enables the use of a wide range of materials, including electroactive materials ranging from metals to organic semiconductors, supporting materials with varying stiffness, and surface functionalization with biomolecules to enhance cell–chip engagement and long-term stability.^39,40^ Given the great versatility of such platforms, MEAs and related neural interfaces have matured into technologies capable of incorporating neuromimetic features that can recapitulate neuronal morphology, as well as electrical and chemical signal processing.

### 3.1 Material composition

The primary requirements for any material to be interfaced with living systems are its biocompatibility and non-toxicity.^41^ Additionally, for successful *in vivo* interfacing of implants with neural tissue, the inflammatory tissue response must be minimized to prevent activation of astrocytes and microglia. These reactive glial cells form a glial scar, *i.e*., an insulating barrier around the probe, thereby reducing the device's performance and long-term stability.^42^

Conventional silicon- and metal-based electronic materials are widely employed in neural interfaces, relying on biocompatible, inert metals (*e.g*., Au, Pt) and oxides (*e.g*., indium tin oxide, TiO_2_, SiO_2_) while taking advantage of well-established high-throughput fabrication processes.^41^ Despite the success of this class of devices, inorganic materials are dry, hard, static, and primarily electronic, whereas neural tissue is wet, soft, dynamic, and ionically active.^41^ As a result, they remain poorly suited to transduce and process the diverse signals that mediate neuronal communication, such as neurotransmitters or ion fluxes. Thus, while inorganic materials provide high conductivity and fabrication reliability, their rigidity and limited surface functionalization capacity constrain seamless integration with neural tissue and limit their ability to emulate a neuronal dynamic environment.^41^

Organic semiconductive materials have been shown to overcome these limitations by mimicking neuronal functions and electrical communication through precise structural design and chemical functionalization.^43^ In this scenario, conductive polymers (CPs) and organic mixed ionic-electronic conductors (OMIECs) have readily become the materials of choice, leveraging the electronic transport of π-conjugated polymers and the ion transport of blended polyelectrolytes.^44,45^ Here, polypyrrole (PPy), polyaniline (PANI), polythiophene (PTh), and poly(3,4-ethylenedioxythiophene) (PEDOT) have emerged as the most promising CPs for neural interfaces.^44^ With its high electrical conductivity and environmental stability, PPy has traditionally been the most widely used CP for biomedical applications. In contrast, although PANI exhibits high electrical conductivity, its application in neural devices is limited by poor cell adhesion and low biocompatibility.^46^ CPs can be synthesized *via* both chemical and electrochemical oxidative routes,^47^ in which strong oxidants (*e.g*., H_2_O_2_) and catalysts, or high voltages (*e.g*., ∼1 V), are required to initiate and propagate polymerization. In neuroelectronic applications, electrochemical polymerization is emerging as a leading method, enabling the localized deposition of CPs on electrode sites, providing high stability in biological environments, allowing simultaneous doping, and facilitating thin-film production by offering greater control over substrate thickness.^44^

**Fig. 3:**
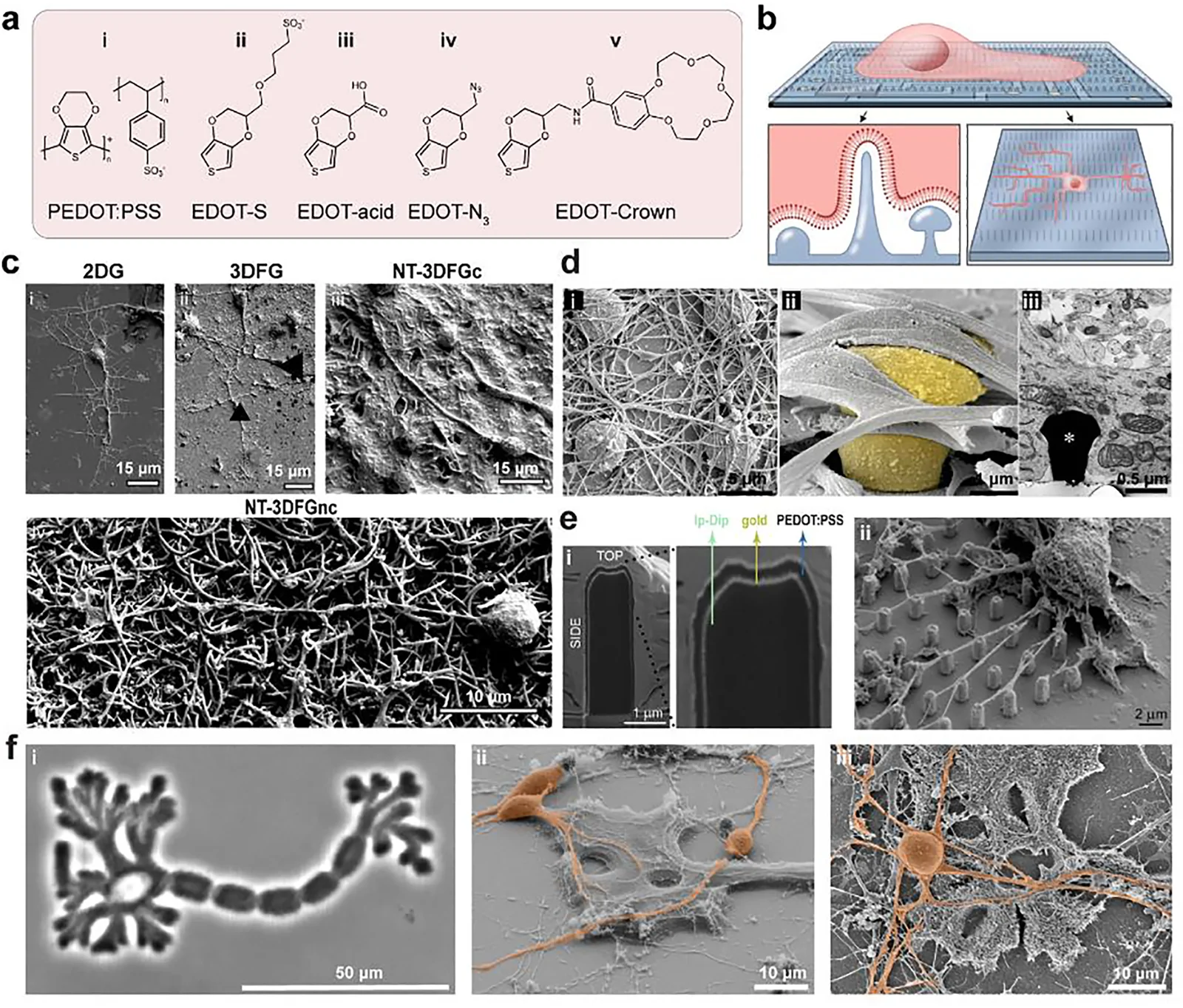
Chemical and structural material design strategies for neural interfaces. (a) Chemical structures of (i) PEDOT:PSS, (ii) EDOT-S, (iii) EDOT-acid, (iv) EDOT-N_3_, (v) EDOT-crown. Chemical structures were generated in ChemDraw. (b) Schematic representation of neurons cultured on the 3D topography. Created in BioRender. Santoro, F. (2026) https://BioRender.com/scnajz7. Some objects in the scheme were generated in Procreate®. (c) Scanning electron micrographs showing the effect of out-of-plane graphene on axonal branching and elongation. Reproduced with permission from ref. 70. Copyright 2022, American Chemical Society. (d) Scanning electron micrographs of neurites on gold mushroom electrodes (i) and (ii), and SEM-FIB cut of a neuron tightly engulfing a gold electrode (iii). Adapted with permission from ref. 71. Copyright 2015 by the authors. Distributed under a Creative Commons CC BY International License (CC BY 4.0; https://creativecommons.org/licenses/by/4.0/). (e) Scanning electron micrograph of the cross section of a 2PP printed micropillar coated with PEDOT:PSS (i) and the pillar-neuronal cell interaction (ii). Adapted with permission from ref. 72 with permission from the Royal Society of Chemistry. (f) Brightfield micrograph of a 2PP printed GelMA/PEDOT blend in a stylized neuron geometry (i) scanning electron micrographs of primary neurons cultured on the 2PP printed structures, false colored in orange (ii–iii). Adapted with permission from ref. 73. Copyright 2026 by the authors. Distributed under a Creative Commons CC BY International License (CC BY 4.0; https://creativecommons.org/licenses/by/4.0/).

Nowadays, poly(3,4-ethylenedioxythiophene):poly(styrenesulfonate) (PEDOT:PSS) (Fig. 3a(i)) represents an exemplary OMIEC, widely used in both *in vitro*^48–50^ and *in vivo*^51–53^ devices for neural recording and stimulation,^54^ due to its high electrochemical stability and ability to lower the impedance of coated metal electrodes.^55^ In the PEDOT:PSS blend, the negatively charged polyelectrolyte PSS acts as a dopant, counterbalancing the positively charged, oxidized, π-conjugated PEDOT. Interestingly, the versatile tools of organic chemistry enable structural modifications of the polymer backbone, endowing specific material properties.^56^ For instance, it is possible to induce self-doping in modified PEDOT bearing a side chain that incorporates a sulfonate group (PEDOT-S) (Fig. 3a(ii)).^57^ Indeed, a PEDOT/S-EDOT copolymer was successfully deposited onto implantable neural probes.^58^ Additionally, by inserting appropriate side-chain moieties, PEDOT can undergo post-functionalization to enable further fine-tuning of material properties.^59^ To this end, different strategies can be followed,^60,61^ for instance, with the insertion of a carboxylic group (Fig. 3a(iii))^62,63^ or an azido moiety at the end of a side chain (Fig. 3a(iv)).^64^ The former readily binds to amine groups, enabling linkage to proteins, peptides, and other biomolecules.^65^ The latter exploits “click chemistry” to bond any type of organic compound bearing an alkyne functionality.^66^ Moreover, the organic nature of CPs makes them amenable to functionalization with a variety of biomolecules, as embedded dopants, adsorbed, grafted, or covalently bonded, thereby enhancing cell adhesion, proliferation, and long-term stability.^67^ The nearly infinite possibilities offered by organic chemistry are instrumental in targeting and mimicking specific neuronal functions. For instance, an interesting opportunity can be provided by modifying PEDOT bonding ion-selective molecules such as crown ethers (Fig. 3a(v)).^68,69^ This class of organic cyclic compounds, composed of alternating carbon and oxygen atoms, can efficiently and selectively bind various metal ions, depending on the size of the chosen crown ether. Therefore, we envision that such a system could potentially emulate the ion-mediated signal transmission at the synaptic level.

Overall, the good biocompatibility, chemical stability, mixed ionic-electronic conduction, softness, tunability, and possibility of functionalization with bioactive molecules make CPs and OMIECs particularly attractive for neural interfaces.^41,54^ Their mixed ionic-electronic conductivity is especially important, as it enables coupling between ionic biological signals and electronic devices. However, their performance depends strongly on dopant chemistry, film stability, swelling behavior, long-term adhesion, and polymerization route, which remain important considerations for neural interface applications. The advantages and limitations are summarized in Table 1.

**Table 1:** Advantages and limitations of materials for neural interfaces

Material class	Advantages	Disadvantages
Silicon- and metal-based inorganic materials: inert metals, silicon, oxides	High conductivity, mechanical robustness, high-throughput fabrication, and compatibility with established microfabrication processes.^41,74^	Poor integration due to a mechanical mismatch between dry, rigid materials and soft and wet neural tissue. Limited capacity for chemical surface functionalization.^41^
Organic CPs: PPy, PANI, PTh, PEDOT	Good biocompatibility and stability, and sufficient electrical conductivity. Chemical tunability and the ability to incorporate dopants or bioactive molecules.^44^	Conductivity, biocompatibility, and cell adhesion vary between materials. Performance depends on the dopant. Limited use in biomedical applications due to low processability and poor mechanical properties.^44^
OMIECs: PEDOT:PSS and its derivatives	Biocompatibility, non-toxicity, and mechanical softness. Mixed ionic-electronic conduction and high electrochemical stability. Chemical tunability. Widely used in neural interfaces.^54^	*In vivo* degradation, poor adhesion to substrates, and lack of standard processing protocols. Stability and performance depend on dopant, underlying substrate, and processing.^54^
Composite materials and hydrogels	Soft with mechanical stiffness similar to that of the tissue, high water content, and tunable mechanical properties. Minimal cytotoxicity and high biocompatibility.^41,75^	Low conductivity (requires conductive fillers or CP). Possible trade-offs between conductivity, mechanics, and stability.^41,75^

### 3.2 Topographical arrangement

A deeper understanding of the mechanisms underlying network formation and synaptogenesis requires a shift in engineering neural interfaces from two-dimensional (2D) to three-dimensional (3D) systems, which can better replicate the brain's complex 3D architecture.^76^ In this regard, a neurohybrid interface should address the complexity of brain and neuronal architectures by structurally mimicking their key features.^77,78^ Furthermore, 2.5D and 3D topographies can enhance the quality of recorded signals by enhancing cell–chip coupling.^35^

The intricate 3D connections that build neuronal networks, and ultimately the brain, are responsive to a plethora of stimuli, including topographical cues.^79^ In particular, neuronal development and neurite elongation are strongly influenced by the environment, including the neighboring cells and extracellular matrix (ECM) molecules that promote the formation of synaptic connections.^80^ Therefore, the design of a neurohybrid platform should comprise 2.5D topographies, such as micro- and nano-pillars, and 3D topographies, such as scaffolds, that neurons recognize as structural counterparts or ECM analogs. Such topographies can act as triggers for neural network modulation, synapse formation, and axon outgrowth (Fig. 3b). Neuronal behavior can be modulated by changes in the environment at the micro- and nanoscale, making neurons sensitive to substrate roughness and porosity.^81^ For instance, pseudo-3D electrodes made of out-of-plane graphene strongly modulate neuronal networks in the early stages of development by influencing growth cone formation and spatial exploration (Fig. 3c).^70^ Furthermore, rough topographies can enhance network connectivity, as demonstrated on etched silicon, where neuronal cultures developed small-world networks.^82^ Interestingly, anisotropic structured surfaces can serve as guidance cues that promote neurite and axon elongation with imposed directionality.^83^ To this end, micro- and nano-patterned grooves with varying aspect ratios of ridge height to valley width have been investigated, demonstrating that neurites can follow elongated paths.^84,85^ In addition, 2.5D and 3D micro- and nano-structured materials of various shapes, such as pillars or cones, are sensed by cells, which recruit focal adhesion proteins and rearrange the cell membrane and actin cytoskeleton at the interface, resulting in engulfment of these structures and tight substrate coupling.^74,86^

Beyond providing instructive cues for neuronal growth and connectivity, 2.5D and 3D topographies are particularly important in neuroelectronics, where an electrical device directly interfaces with cells, tissues, or entire organs.^35^ These topographies can improve cell–chip coupling by promoting membrane wrapping and engulfment of structured electrodes. The intimate contact brings recording sites closer to neurons, increases the effective electrode surface area, and reduces impedance, thereby improving the quality of electrophysiological recordings and the signal-to-noise ratio (SNR).^35^ For example, mushroom-shaped gold protrusions were designed to emulate dendritic spines (Fig. 3d).^71,87^ Their enhanced cell–chip coupling and low invasiveness, *i.e.*, ability to preserve membrane integrity compared to nano- and micropillars, have inspired numerous follow-up studies.^88–90^ Furthermore, by leveraging a variety of microfabrication techniques,^91,92^ it is possible to engineer electrodes that mimic the dendritic spine shapes during synaptogenesis, such as filopodia and mushroom spines.^93^ Filopodia-like structures can be realized as micro- and nano-pillars of both inorganic^94,95^ and organic^96^ materials, together with conductors^97,98^ and dielectrics.^83,99^ Notably, organic semiconductors have also been employed for the fabrication of micro-pillars. For example, PEDOT has been extensively investigated for integration onto 2.5D and 3D microstructures for neural interfaces, primarily *via* electrodeposition on various substrates, such as mushroom-shaped gold microelectrodes^50^ and flexible PDMS-based micropillars.^100^ Interestingly, the innovative 2-photon polymerization (2PP) lithography has also been used to realize pillars of different aspect ratios, electrodepositing PEDOT:PSS, both inside hollow structures^101^ and as a coating on micropillar arrays (Fig. 3e).^72^ Moreover, 2PP enables patterning of more complex structures, such as scaffolds that support the formation of 3D neuronal networks in a highly customizable and controlled environment.^102^

As illustrated by the examples above, nano- and micro-topographies enhance neuronal adhesion and guidance, and electrophysiological recording quality through tight cell–electrode coupling.^103^ Functionalization of these structures with bioactive molecules can further modulate cellular processes.^103^ However, increasing topographical complexity can introduce challenges related to reproducible fabrication and scalability.^103^ Moreover, the rigid structures can potentially introduce mechanical stress and tissue damage at the cell–material interface.^103^ Therefore, optimal designs must balance enhanced coupling with minimal invasiveness, particularly for long-term neural interfaces. Combining these architectures with soft materials, such as hydrogels, can reduce mechanical mismatch with neural tissue, as discussed in the following section. The advantages and limitations of different topographies are summarized in Table 2.

**Table 2:** Advantages and limitations of topographies for neurohybrid interfaces

Topographical arrangement	Advantages	Disadvantages
2D planar interfaces	Simple fabrication, reproducibility, compatibility with conventional MEAs.^74^	Limited mirroring of 3D brain architecture. Weak cell–chip coupling and low recorded signals.^74^
Rough or porous surfaces	Enhanced neuronal adhesion, modulation of neurite growth and network connectivity.^70,74,82–84,103,104^	Limited control over cell placement. Potential variability in surface properties.^74^
Micro- and nanopillars, cones, and mushroom-shaped electrodes	Promote tight neuronal engulfment and protein recruitment at the interface. Enhanced tight cell–chip coupling and recorded electrical signal. Possible functionalization with bioactive molecules.^71,72,83,86,87,94,95,99,100,105^	Possible mechanical stress or membrane damage, depending on geometry and stiffness. Fabrication and scalability challenges. Stiff unless combined with hydrogels, and mostly non-conductive.^88,105^
Conductive scaffolds	Support 3D neuronal networks, cell infiltration, and ECM-like organization.^72,74^	Diffusion constraints, reproducibility, and integration with electrodes is challenging.^74^
Composite materials and hydrogels	Soft, wet, with tunable mechanical and adhesive properties. Excellent for 3D cell support and neural modulation. Complex structures can be produced with 2PP lithography. Drug release possible *in vivo*.^41,73,75,106–108^	Mechanical stability and long-term performance require optimization. Sensitive to temperature and humidity changes. Possibly challenging scalability.^75,103^

### 3.3 Conductive hydrogels for neural interfaces

Composite materials could further expand the options for realizing neuromimetic interfaces. Within this class, hydrogels are among the most incorporated phases that, due to their high-water content and tunable mechanical properties,^41^ can be engineered to closely approximate the mechanical characteristics of native neural/brain tissue, making them ideal scaffolds for materials that can reduce tissue responses at the electrode–tissue interface.^41,108^ Interestingly, the typical Young's modulus range of hydrogels (1–100 kPa)^109^ closely matches the mechanical properties of the brain. While polymeric hydrogels improve mechanical compliance, the inherently low electronic conductivity limits their ability to transmit electrical signals efficiently.^41^ To overcome this challenge, conductive polymers have been incorporated into hydrogel matrices, resulting in a class of hybrid materials known as semi-interpenetrating networks (semi-IPNs).^41^ These composites provide electronic functionality while retaining the mechanical softness of hydrogels, making them well-suited for neural interface applications. For instance, PEDOT nanoparticles incorporated into a chitosan/gelatin (Cs/Gel) scaffold yielded a conductive substrate with improved biodegradability, mechanical, and electrical properties, and exhibited enhanced adhesion and neurite growth in cultured neuronal cells.^110^ A key advantage of hydrogel scaffolds is their 3D structure, which more closely mimics the physiological environment of cells than traditional 2D culture systems.^111^ Porous scaffolds not only provide structural support for cell growth but also enable cell infiltration, a crucial cue for developing realistic neural tissue. Building on the importance of a 3D environment, a 3D-printable conductive hydrogel based on PEDOT:PSS was developed, offering both excellent structural support and high electrical conductivity. When integrated with encapsulated dorsal root ganglion (DRG) neurons, the scaffold exhibited minimal cytotoxicity and significantly enhanced neural cell differentiation under electrical stimulation (ES).^107^ In addition, photocurable hydrogels, such as the methacrylated gelatin (GelMA), can be employed in 2PP lithography to realize complex soft scaffolds^106^ and can be engineered to be conductive upon the introduction of functional fillers.^112^ Recently, a 2PP photocurable GelMa/PEDOT:PSS blend has been developed and demonstrated the possibility to print neuron-like structures at the microscale (Fig. 3f).^73^ These findings highlight the promise of 3D conductive hydrogels as multifunctional bioelectronic platforms capable of modulating cell behavior *via* electrical stimulation.

### 3.4 Neuron-inspired soft interfaces for *in vivo* integration

Design principles developed for *in vitro* platforms have also been translated to *in vivo* devices, where the use of soft, conformable materials is a key requirement for a stable chip–brain connection. From brain organoids^113^ to the whole brain, novel device architectures are increasingly drawing structural inspiration from the neural tissue itself. A notable example is a recent *NeuE* probe, designed to recapitulate the mechanical and structural features of neurons, demonstrating seamless interpenetration in the live murine brain.^78^ Similarly, the flexible *NeuroRoots* implant, inspired by axons, demonstrated high-quality chronic electrophysiological recordings in freely moving rats.^114^ Furthermore, PEDOT:PSS was selected as an electrode coating to lower the impedance, underscoring the wide range of applications for CPs. Additionally, micro- and nanostructured electrodes with protruding pillars were also implemented in *in vivo* implants, yielding devices that combine flexibility with improved cell–chip coupling.^115,116^ Notably, PEDOT:PSS was the material of choice for the neuropillar array, where the soft properties of PDMS-based micropillars are combined with the mixed ionic-electronic conduction of the CP coating.^117^ In addition to CPs, hydrogels are increasingly gaining prominence as neural interfaces for implants due to their mechanical properties that closely resemble those of brain tissue, improved cell adhesion, and high versatility in chemical composition and tunable performance.^118^ Importantly, hydrogels also offer the potential for closed-loop feedback control of on-demand treatment of pathologies, using hydrogel matrices to release drugs in response to real-time electrophysiological inputs.^119^

## 4. Organic neuromorphic biointerfaces

**Fig. 4:**
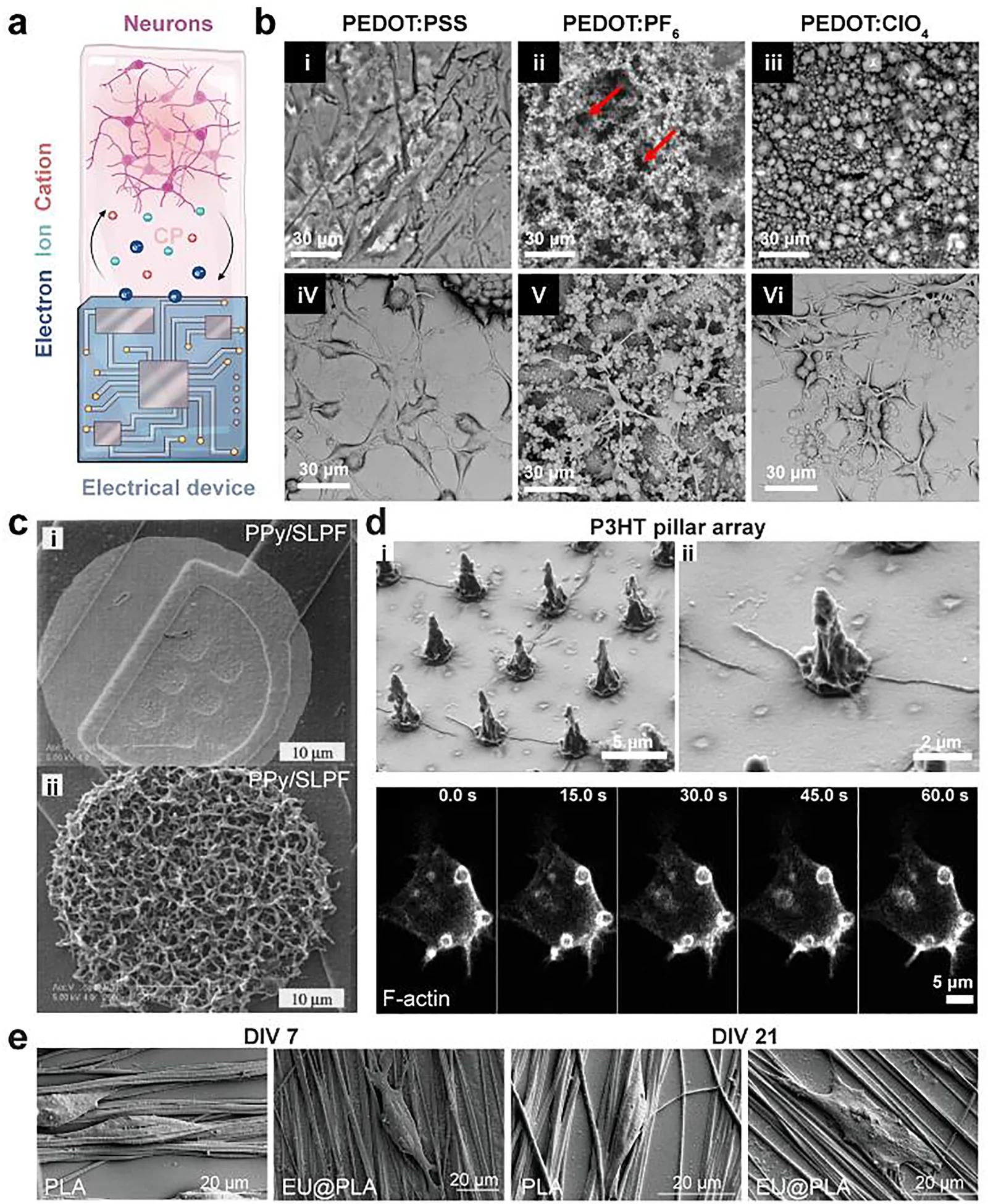
Organic semiconductors as neural interfaces. (a) Schematic representation of biopolymers as neural interfaces. https://biorender.com/t0bzubm. Some objects in the scheme were generated in Procreate®. (b) Scanning electron micrographs of (i) PEDOT:PSS, (ii) PEDOT:PF_6_, and (iii) PEDOT:ClO_4_ electrodeposited on a platinum plate and neuronal B35 cells cultured on the corresponding polymers (iv–vi). Reproduced with permission from ref. 122. Copyright 2021 by the authors. Licensee MDPI, Basel. Distributed under the terms and conditions of the Creative Commons Attribution (CC BY) license (https://creativecommons.org/licenses/by/4.0/). (c) Scanning electron micrographs of PPy electrodeposited on gold electrodes at increasing deposition times. Adapted with permission from ref. 123 with permission from the Royal Society of Chemistry. (d) Scanning electron micrographs of micropillar array based on P3HT polymer (i and ii, top), and formation of F-actin rings around the pillars over time (bottom). Reproduced with permission from ref. 124. Copyright 2021, American Chemical Society. Distributed under a Creative Commons CC BY International License (CC BY 4.0; https://creativecommons.org/licenses/by/4.0/). (e) Scanning electron micrographs of neuronal cell cultured on PLA and eumelanin-coated fibers at different days *in vitro* (DIV). Reproduced with permission from ref. 125. Copyright 2023 by the Authors. Advanced Materials Interfaces published by Wiley-VCH GmbH. Distributed under a Creative Commons CC BY International License (CC BY 4.0; https://creativecommons.org/licenses/by/4.0/).

The previous section underscores the importance of material selection in developing interfaces that bridge neuromorphic bioelectronics and living biological systems from both chemical and structural perspectives. It is imperative that substrates in neural interfaces meet the biological, mechanical, and electrochemical requirements of host living tissues, enabling safe neuron-technology interaction and a smooth exchange of electrophysiological information (Fig. 4a).^46^ As shown, increasing attention has been directed toward conductive soft organic materials that closely match the mechanical and electrical properties of brain tissue, representing a promising route in the development of neuromorphic interfaces.^120^ The versatility of electrosynthesis in the presence of different doping ions makes CPs ideal for optimizing properties for neuromorphic interfaces and tuning them to specific application requirements.^121^

Notably, it has recently been shown that the choice of dopant in CPs-based neural interfaces is critical, as it directly influences the biocompatibility, electrochemical, and mechanical properties of the resulting substrate.^41,122^ For instance, the viability of rat neural stem cells (NSCs) on PPy substrates was found to be highly dependent on the doping ion.^122^ While dodecylbenzenesulfonate (DBS) supports high NSCs viability, a significant reduction in survival was observed on PPy doped with tosylate (TsO^−^), perchlorate (ClO_4_^−^), or chloride (Cl^−^). Similarly, differences in the viability of rat neuronal cells cultured on PEDOT films doped with PSS^−^, ClO_4_^−^, or hexafluorophosphate (PF_6_^−^) were observed, with ClO_4_^−^ yielding the highest viability.^122^ Biological responses to CP substrates depend not only on chemical composition but also on dopant size, which strongly influences surface morphology.^46,122^ For instance, PEDOT doped with small counterions, such as ClO_4_^−^, paratoluene sulfonate (pTS), or benzenesulfonate, yields rougher, compact, porous films that are topographically favorable for biological tissue and enhance cell adhesion. In contrast, doping with larger ions, such as PSS^−^, results in smoother, non-uniform morphologies that generally reduce cell adhesion (Fig. 4b).^122^ Finally, altering topographical cues on substrates through dopant selection can promote neural development, as neural outgrowth is strongly linked to dopant type,^126^ with rougher surfaces supporting more developed and highly branched axon networks.^126^

The biocompatibility and cell interaction of conductive polymers can be further enhanced by incorporating non-doping, bioactive molecules.^126^ For instance, embedding nerve growth factor (NGF) into PPy films promoted adhesion and neurite extension of rat pheochromocytoma (PC12) cells.^127,128^ This approach has also been applied to PEDOT substrates, showing comparable improvements in both cell adhesion and neurite outgrowth.^127^ Similarly, PPy, combined with a silk-like polymer with fibronectin functionality (SLPF) and laminin fragment CDPGYIGSR, improved neural interfacial contact and neurite outgrowth (Fig. 4c).^123^ The subsequent study demonstrated selective cell adhesion, with glial cells preferentially attaching to a PPy/SLPF-coated substrate, and neuroblastoma cells favored the PPy/CDPGYIGSR substrate.

Furthermore, optically active CPs, such as poly[2,6-(4,4-bis-(2-ethylhexyl)-4*H*-cyclopenta [2,1-*b*;3,4-*b*′]dithiophene)-*alt*-4,7(2,1,3-benzothiadiazole)] (PCPDTBT)^129^ and regioregular P3HT,^124^ expand the functional repertoire of CP-based interfaces by transducing light into electrical current, thus enabling non-invasive optical actuation of neuronal activity. For instance, flat and nanostructured P3HT-based substrates, which combine optical stimulation with topographical cues, demonstrated excellent biocompatibility when neurons were cultured on them, and, upon light stimulation, the material promoted neurite outgrowth and enhanced neuronal development (Fig. 4d).^124,129^ This study further demonstrated light-mediated inhibition of neuronal activity, highlighting the potential of this approach for treating pathologies characterized by neural hyperactivity.

In addition to their favorable electrochemical and biological properties, the soft and flexible nature of CPs provides a critical advantage for neural interface applications.^46,130^ Despite their promising properties, conductive polymers alone may not always fully satisfy the combined requirements for neural interface applications. Consequently, extensive research has focused on developing composite materials to better address these demands by combining mechanical compliance, electrical performance, long-term stability, and biocompatibility for optimized neural recording and stimulation.^108^ Imparting electrical conductivity to polymeric biomaterials demonstrates significant potential to engineer scaffolds that are highly interactive with the biological environment, particularly at neural interfaces.^108^ Among naturally occurring polymers, polysaccharides and proteins modified with conductive elements represent a class of bio-derived materials capable of supporting neuronal cell interfacing.^108^ Notably, melanin, the ubiquitous pigment found across diverse living organisms, has recently emerged as a promising candidate for organic bioelectronics applications.^131^ Eumelanin, biogenetically derived from the oxidative polymerization of 5,6-dihydroxy-indole (DHI) intermediates and 5,6-dihydroxyindole-2-carboxylic acid (DHICA), is particularly attractive due to its intrinsic biocompatibility and mixed ionic-electronic conductivity.^132,133^ In addition, eumelanin and its analogues, *e.g*., polydopamine (PDA), exhibit adhesive properties *via* both covalent and non-covalent interactions, the ability to chelate metal ions, UV absorption, antioxidant properties, and redox activity.^134,135^ Thin films can be realized *via* solution processing and spin coating,^136^ which have been shown to enhance *in vitro* Schwann cell attachment and growth and to promote neurite extension in PC12 neuronal cells.^137^

Eumelanin-based materials have also shown great promise as components of topographically instructive scaffolds. For instance, eumelanin-coated poly(lactic acid) (PLA) fibers were investigated as potential substrates for culturing human-derived SH-SY5Y neuroblastoma cells (Fig. 4e).^125^ While both coated and uncoated PLA fibers supported cell growth and proliferation, the eumelanin-coated ones significantly enhanced directionality guidance and modulated neuronal behavior. The coating was essential for promoting SH-SY5Y cell spreading, alignment along the substrate's main axis, and neuronal differentiation.^138^ Furthermore, the blending of melanin with poly-3-hydroxybutyrate (PHB) yielded a novel, semiconductive, non-toxic, and biodegradable material with a distinct surface topography. Since this blend was compatible with electrospinning, it enabled the fabrication of a 3D nanofibrous polymeric network (scaffolds) with poroviscoelastic properties that match those of neural tissue, enhancing the adhesion and growth of mouse DRG and motor neurons.^139^ When combined with traditional CPs, eumelanin can enhance their mechanical properties, therefore complementing CPs’ excellent electrical conductivity. Eumelanin–PEDOT:PSS (Eu–PH) films exhibited enhanced water stability and improved adhesion of PEDOT:PSS, which is often prone to cracking and delamination from the underlying substrates.^140^ A melanin-like polymer, polydopamine melanin (PDAM), has also been explored as a dopant for PEDOT (PEDOT/PDAM).^138^ PDAM shares a similar chemical structure and origin with natural melanin, offering additional benefits to PEDOT, including enhanced electrochemical and mechanical stability. PEDOT/PDAM also proved to be a suitable substrate for culturing human pluripotent stem cell (hiPSC)-derived neural progenitors, supporting their proliferation and promoting neuronal network formation. Moreover, it significantly reduced the impedance of commercial MEA platinum electrodes, thereby improving the quality of neuronal recordings, demonstrating its efficiency as a neural electrode material.

## 5. Life-like interfaces through the incorporation of membranes, vesicles, and synthetic cells

Next to protein- and peptide-based coatings, lipids are another important class of biomolecules. As the major components of cellular membranes, they can also provide a potentially biocompatible interface for implants. In addition, lipids are amphiphilic molecules capable of self-assembling into lipid bilayer membranes, even outside living cells, and can coat surfaces as so-called supported lipid bilayers (SLBs). In a neural-interface context, titanium surfaces covalently tethered with PC12–cell–membrane-derived lipid bilayers showed resistance to plasma-protein fouling, blood components, and bacterial adhesion, while reducing interactions with astrocytes and macrophages.^141^ At the same time, these neuronal cell-derived lipid bilayers promoted neuronal adhesion, neurite outgrowth, and neuronal activation, illustrating how lipid coatings can provide both antifouling and cell-instructive functions for neuro-implantable devices.^141^ However, through their dynamic voltage responses and incorporation of additional molecules, SLBs contribute more than a simple capacitive component to the electrical response. For example, SLB-coated PEDOT:PSS electrodes exhibit irreversible interactions with serotonin (5-HT) and dopamine (DA), enabling Pavlovian associative learning *via* a change in device conductance^142^ and dynamic dedoping by cations injected with high-frequency electrical pulses.^143^ These examples show the potential of SLBs to contribute both to enhanced biocompatibility and neuromorphic function. However, integration with existing electronics requires stable and low defect densities in SLB.^144^ Notably, very low defect densities with specific resistances exceeding 10^6^ Ω cm^2^ droplet-interface lipid bilayers have been demonstrated,^145^ showing the potential of more complex geometries and lipid material systems. Going beyond two-dimensional SLBs, titanium implants were functionalized with cell-derived 3D extracellular vesicles for enhanced osseointegration.^146,147^ However, to the best of our knowledge, such attempts have not yet been demonstrated for neurohybrid interfaces. It seems clear, however, that increasingly complex lipid systems that promise to mimic living cells more closely would potentially provide a natural implant interface.^120^ Indeed, so-called “synthetic cells” based on spherical lipid membrane vesicles could enable 3D integration with the compliant mechanical and electrical properties of biological cells.^148^ Synthetic tissue-like networks have been shown to assemble into 3D structures on glass substrates^149^ and can be incorporated into agarose-based hydrogels.^150^ Lipid vesicles and synthetic cells have also been interfaced with field-effect transistors,^151^ although integration with organic bioelectronics has not yet been achieved. Taken together, these developments highlight the potential of interfacing neural implants with synthetic cell networks exhibiting neuromorphic functionality.^152^

## 6. Neuroinspired living electronics

**Fig. 5:**
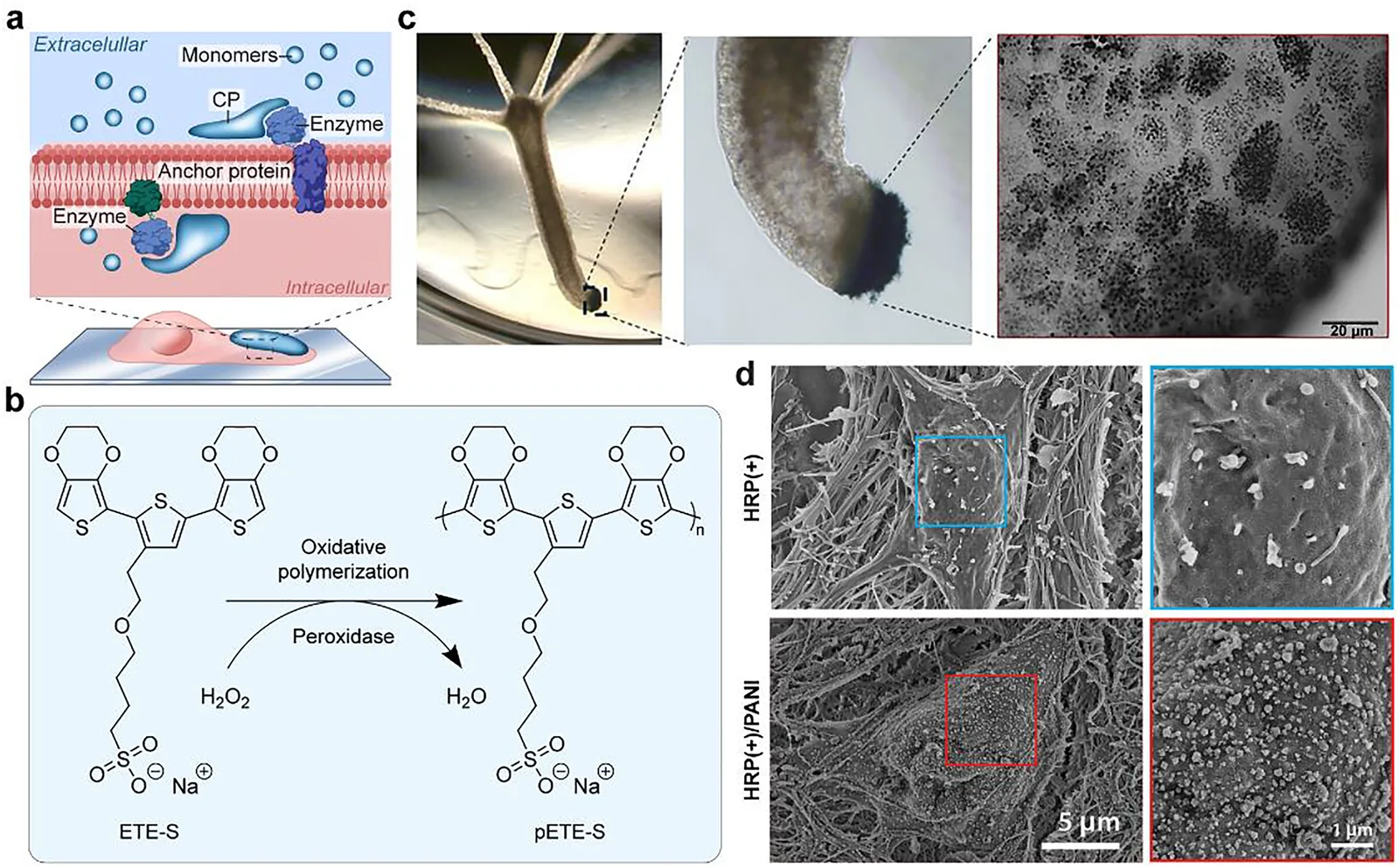
*In situ* polymerization of conductive organic materials. (a) Schematic illustration of *in situ* formation of conductive polymer (CP) intra- or extracellular by membrane-anchored enzymes. Scheme was generated in Procreate® and imported in BioRender for final modifications. Santoro, F. (2026) https://BioRender.com/8zcbcs2. Scheme was inspired by ref. 7 and 8. (b) Oxidative polymerization process of ETE-S to pETE-S by peroxidases in the presence of hydrogen peroxide. Chemical structures were generated in ChemDraw. (c) ETE-S polymerization in *Hydra vulgaris*, initiated by endogenous peroxidase activity and exogenous hydrogen peroxide. Reproduced with permission from ref. 154. Copyright 2025, the author(s). Advanced Materials Interfaces is published by Wiley-VCH GmbH. Distributed under a Creative Commons CC BY International License (CC BY 4.0; https://creativecommons.org/licenses/by/4.0/). (d) Scanning electron micrographs of HRP-expressing neurons with and without 30 minutes of PANI deposition. Reproduced with permission from ref. 8. Copyright 2023 the authors, some rights reserved; exclusive licensee American Association for the Advancement of Science. No claim to original U.S. Government Works. Distributed under a Creative Commons Attribution License 4.0 (CC BY 4.0; https://creativecommons.org/licenses/by/4.0/).

As outlined in the previous sections, CPs offer significant advantages as materials for neural interfaces. By acting as neuroprosthetics or neuromorphic materials, they pave the way toward seamless integration between electronic devices and biological tissues. A further conceptual advance lies in the *in situ* polymerization of CPs directly within living neural tissue (Fig. 5a) to develop so-called living neuroelectronics. Direct polymer formation within tissue has the potential to minimize the mismatch between the biotic and abiotic domains, thereby improving long-term integration and stability.^153^ This strategy offers a route toward living electronic materials that can evolve, adapt to their environment, and respond to stimuli, potentially supporting tissue regeneration and restoration of lost electrical functionalities. In this section, we discuss requirements and strategies for *in situ* polymerization, illustrated by examples from the literature. We also address targeted approaches for integrating living electronics at cellular and compartment-specific levels.

### 6.1 Requirements for *in situ* polymerization

A prerequisite for *in situ* polymerization is the biocompatibility of all reactants and of the polymerization reaction itself, to avoid unwanted toxicity. The polymerization should proceed under physiological conditions, particularly with respect to solubility in aqueous systems, pH, and temperature.^7,155^ The reaction should occur without undesirable side reactions in the presence of metabolites or other compounds naturally present in the tissue. To achieve controlled and localized polymerization, endogenous or exogenous stimuli, such as redox reactants,^123,156^ enzymes,^157^ or light,^158^ can be used. In addition, polymer properties should be tailored to match the mechanical, electrical, and biological properties of neural tissue.^7^ While natural building blocks, such as amino acids, nucleotides, or monosaccharides, exhibit biological recognition and functionality, they often lack electrical conductivity and tunability.^7,159^ In contrast, artificial monomers are gaining increasing interest, as they can be designed to synthesize CPs with customized properties, such as stiffness, elasticity, biocompatibility, degradability, and conductivity.^7^

### 6.2 Conductive polymers for *in situ* polymerization in living tissue

As a widely used CP in neural interfaces, PEDOT was employed in early *ex vivo* and *in vivo* studies to investigate whether conductive polymers could be formed directly within living neural tissue and integrate with it. These studies investigated the effect of an electrochemically deposited PEDOT cloud around an implanted electrode.^160,161^ In one *ex vivo* study, the direct integration of PEDOT filamentous network into mouse brain slices was demonstrated, with filaments extending up to 1 mm into the tissue. This network bypassed the insulating glial scar and increased the electrode's effective surface area.^160^ Interestingly, when a similar polymerization strategy was applied *in vivo* in rats, no evident behavioral disruptions were observed, further highlighting the potential of directly growing CP within living systems.^161^ Despite these promising findings, this groundbreaking approach has important limitations. These include the potentially adverse effects of high currents required to electrochemically polymerize CPs around the implanted electrode on the surrounding brain tissue, as well as secondary scarring around the PEDOT cloud. To mitigate these adverse effects and achieve truly seamless integration, different polymerization strategies and materials should be explored.

In this context, advances in CPs design have enabled the development of self-doped polythiophenes, offering an attractive alternative to heterogeneous CPs by eliminating the necessity for an external dopant. An example is a thiophene trimer, developed as a new class of monomers (ETE), composed of two outer 3,4-ethylenedioxythiophene (EDOT) moieties and a central thiophene bearing a modifiable side chain. Such a design lowers oxidation potential and water solubility.^162,163^ Interestingly, the presence of a hydroxyl side chain allows the introduction of various functionalities, ranging from self-dopant groups to anchoring structures. Ionic groups such as sulfonic acid (ETE-S, Fig. 5b)^162,164^ or carboxylic acid (ETE-COONa)^157^ act as self-dopants, while hydrophobic oleyl groups act as anchors that can be inserted into the membrane bilayer of cells, enabling stable localization.^165^ These ETE-based monomers have been successfully polymerized across diverse biological systems, as discussed in the following section.

### 6.3 *In situ* polymerization of self-doped CPs across living systems

Self-doped CPs were first demonstrated for *in situ* polymerization in living plants, where the vascular architecture provided a natural template for conductive polymer formation.^162,166^ In *Rosa floribunda*, ETE-S polymerization enabled the formation of long-range conducting wires along the xylem,^162^ mediated by endogenous peroxidases and hydrogen peroxide (H_2_O_2_).^162,166^ The resulting ETE-S polymer showed higher conductivity than PEDOT-S and maintained it over long distances.^162^

Following the fundamental work in plants,^162,166^*in vivo* studies in *Hydra vulgaris*, an invertebrate with a simple nervous system, demonstrated ETE-S polymerization driven by endogenous peroxidase-like enzymes.^164,167^ As a result, electronically conductive and electrochemically active microdomains were formed within the animal tissue and secreted mucus. Exogenous H_2_O_2_ further enhanced polymerization within these domains (Fig. 5c).^164^ Subsequent *in vivo* studies suggested that the ETE-S monomer itself can modulate Hydra's behavioral responses and electrical activity in a calcium-dependent manner.^154^ Neuromodulatory responses were also observed after backbone modifications to three EDOT moieties (EEE-S) and the side-chain variation to a trimethylammonium group (ETE-N).^154,167^ Together, these findings highlight the potential of ETE-based materials as neuromodulators or electronic interfaces, depending on their configuration and polymerization state.

In another approach, modification of the ETE-monomer with a 2-ethoxyacetic acid sodium salt side chain (ETE-COONa) enabled the *in vivo* fabrication of soft substrate-free electrodes within the nervous systems of small animal models.^157^ The prepolymer mixture containing the ETE-COONa monomer, HRP as a polymerization catalyst, oxidase enzymes for local *in situ* conversion of endogenous metabolites (glucose/lactate) to H_2_O_2_ in the presence of O_2_, polyelectrolyte with counterions for crosslinking, and surfactant for stabilization was injected into the tissue. In zebrafish (*Danio rerio*), polymerization and subsequent gelation did not induce changes in swimming behavior or structural brain damage. Furthermore, the gel electrode mixture was used as an extended electrode in the medicinal leech (*Hirudo medicinalis*) to stimulate the connecting nerve and subsequently induce muscle contractions.^157^

Furthermore, in a recent study,^165^ functionalization of the ETE monomer side chain with hydrophobic oleyl moieties (O) and hydrophilic poly(ethylene glycol) (PEG) yielded a water-soluble ETE-PEGO trimer that enabled *in situ* polymerization on the membrane of living neuronal cells derived from rats. ETE-PEGO anchored to the outer leaflet of the membrane through spontaneous insertion of the oleyl moiety. Subsequently, it was polymerized at the cell surface by adding ETE-S monomer, horseradish peroxidase (HRP), and H_2_O_2_.^165^ This approach enables localized polymerization in neuronal systems of mammals by anchoring monomers directly at the plasma membrane. However, its dependence on exogenous HRP and H_2_O_2_ indicates that further optimization is needed to achieve precise polymerization within neural tissue.

Taken together, ETE-based strategies demonstrate successful polymerization across diverse biological systems, including mammalian cells. However, translating these approaches into living neural tissue requires improved control over polymer localization, diffusion, enzymatic activity, metabolite availability, and H_2_O_2_ production. Targeted *in situ* polymerization approaches that can selectively deposit conductive polymers at defined cellular and subcellular compartments, while preserving biocompatibility, are therefore key requirements for truly living neuroelectronics.

### 6.4 Cell-based *in situ* polymerization for living neuroelectronics

Based on the targeted cellular compartment, targeted *in situ* polymerization approaches can be broadly classified as intracellular, extracellular, and cell-surface-localized.^7^ Intracellular polymerization requires membrane-permeable, biocompatible monomers, whose polymerization is triggered by a particular stimulus. The limitations of this approach are inefficient enrichment of intracellular monomers and potential interference with native intracellular chemistry, which may lead to off-target effects or cytotoxicity.^7^ By contrast, the extracellular polymerization approach minimizes adverse reactions by preventing the reactants from crossing the cell membrane. This approach allows monomers to diffuse into the surrounding tissue and form a polymer outside cells, making it suitable for applications such as *in vivo* electrode assembly.^7^ However, monomer diffusion range and polymerization kinetics must be precisely controlled to achieve localized polymerization. For cell–surface-localized polymerization, initiators, catalysts, monomers, or polymers should be anchored to the cell surface to enable targeted stimulation of specific cells.^7^ This anchoring can be achieved through electrostatic interactions, chemical bonding, or genetic engineering.^7^ As this type of targeted *in situ* polymerization enables cell-specific modulation and stimulation, it represents one of the most relevant strategies for achieving truly living neuroelectronics. In the following section, we discuss emerging approaches for cell-specific material assembly based on genetic engineering.

### 6.5 Genetically targeted chemical assembly for cell-specific living electronics

A central challenge for *in situ* polymerization in living neural tissue is the selective delivery and formation of materials at defined cell types or compartments.^7^ These include neurons, dendrites, or synapses, where precise material placement is required for stimulation or signal detection. To overcome this limitation, genetically targeted chemical assembly (GTCA) has been developed as an approach that utilizes living cells to fabricate CPs directly at the targeted cell surface.^168^ In this approach, cells are genetically modified to express recombinant proteins or enzymes on the outer side of plasma membrane. These surface-expressed components can then initiate *in situ* polymer deposition, enabling spatially defined material formation with desired functionality (Fig. 5a).

Examples include the genetically encoded biotin-binding protein streptavidin (SA)^169^ and photosensitizers, such as the mini singlet oxygen generator (miniSOG).^170^ In the first example, pre-synthesized biotin-coated gold nanoparticles can be uniformly aggregated at the cell surface of streptavidin-expressing neurons.^8^ In the case of miniSOG, targeted illumination of cells expressing the photosensitizer locally generates reactive oxygen species (ROS), which initiate oxidative polymerization. Notably, this method enables dynamic cell patterning by switching illumination on and off.^8,163^*In vitro* studies using iterative PANI and PDAB optogenetic polymerization in rat cortical neurons demonstrated a stepwise modulation of membrane capacitance and excitability.^158^

An advanced method for inducing oxidative polymerization employs membrane-targeted enzymes as catalytic reaction centers that trigger a one-time reaction. Genetically encoded membrane-targeted enzymes can mediate various reaction types, including redox reactions by oxidoreductases, functional group transfer by transferases, hydrolysis by hydrolases, bond cleavage by lyases, isomerization by isomerases, and covalent linkage by ligases.^171^

Peroxidases, a subclass of oxidoreductases, catalyze the polymerization of both insulating and conductive materials, as demonstrated in various reports.^8,168^ Two widely studied examples are ascorbate peroxidase (APEX2)^172^ and HRP.^8,168^ In the first GTCA approach, APEX2 was used to polymerize an aniline dimer to conductive polyaniline (PANI) or 3,3′-diaminobenzidine (DAB) to form the insulating poly(3,3′-diaminobenzidine (PDAB) in mammalian primary hippocampal neurons, *ex vivo*, and in living mice.^168^ In contrast to PDAB, which decreases capacitance, PANI increases it, with further enhancement achieved by doping with *para*-toluenesulfonic acid. Further *in vivo* experiments in the nematode *Caenorhabditis elegans*, which assessed free-moving behavior, showed that PANI modulates animal behavior by altering membrane capacitance.^168^ In the second GTCA approach,^8^ HRP was used because of its faster kinetics and greater selectivity than APEX2.^173^ HRP-expressing primary rat hippocampal neurons showed outer plasma membrane-specific activity, which led to a decreased concentration of cytotoxic H_2_O_2_. Dense polymer clusters composed of PANI or PDAB did not affect the viability of HRP-expressing neurons (Fig. 5d).

Although peroxidases enable targeted polymerization, the requirement for exogenous H_2_O_2_ remains a limitation to biocompatibility. Recently, genetically engineered Laccase (LaccID) that catalyzes the same reaction as peroxidase, using molecular oxygen instead of H_2_O_2_, was engineered.^174^ A comparison between surface-targeted APEX2, HRP, and LaccID in mammalian human embryonic kidney 293 cells (HEK293T) revealed that both peroxidases exhibited intracellular activity at the cell surface and endoplasmic reticulum (ER) lumen, as well as a significant pool of intracellularly trapped enzyme. At the same time, LaccID was exclusively active at the cell surface. In subsequent steps, a transgenic *Drosophila melanogaster* fruit fly expressing LaccID at the axon termini of neuromuscular junctions in the ventral nerve cord (VNC) was generated.^174^ LaccID-expressing neurons were able to polymerize 3,3′-diaminobenzidine (DAB) in a peroxidase-free environment, showing improved delineation of the plasma membrane compared to APEX2-based labeling. Hence, LaccID is a promising H_2_O_2_-free alternative to peroxidases for *in vivo* polymerization. However, the catalytic activity of LaccID requires further optimization to match or exceed that of peroxidase. Further modifications should improve LaccID stability in complex biological environments containing serum, halides, and thiols, such as those found in living brain tissue, where LaccID activity remains limited. Improving LaccID efficiency will likely require further rounds of directed evolution to enhance enzyme activity under physiological conditions and increase catalytic activity in desired polymerization reactions. Cell-specific or inducible promoters could provide spatial and temporal control over polymerization across desired cell types. In parallel, alternative native or engineered enzymes for polymerization that are selectively expressed in the targeted cell type should be explored to reduce the need for extensive genetic manipulation of cells in biomedical applications. Finally, degradable additives, including those with catalytic centers, could be incorporated to support polymerization while minimizing adverse effects. Overall, improving the specificity and efficiency of LaccID-mediated polymerization remains a central challenge for *in vivo* applications.

## 7. Perspectives and conclusions

Engineering neural interfaces that seamlessly integrate with neural tissue and realizing living electronics require a paradigm shift in device and material design that accounts for the brain's multiscale architecture, spanning from higher-order brain organization down to neural circuits and synapses. Beyond efficient electrical cell–chip coupling, next-generation neurohybrid interfaces should draw inspiration directly from neurons. True biological integration will require the recapitulation of key neuronal features, including conductivity, morphology, mechanical softness, and structural and functional adaptability. Material choice lies at the core of this shift, with organic semiconductors being particularly suitable for effective electrochemical neural coupling, owing to their mixed ionic-electronic conduction, biocompatibility, and nearly infinite molecular tunability.

State-of-the-art microfabrication technologies have demonstrated the potential to replicate key morphological features of neurons, such as high-curvature dendritic spine geometries and complex axonal trajectories. These biomimetic topographies represent an important strategy to guide neurite and axonal growth, promote network formation, and further enhance cell–chip engagement. In parallel, recent efforts in chemistry and materials science have yielded a portfolio of soft materials capable of mimicking the mechanical properties of neural tissues and creating 3D scaffolds and constructs. Combining biomimetic micro- and nanostructures with soft materials such as hydrogels and organic mixed ionic-electronic conductors may help reduce mechanical mismatch and improve chronic tissue integration.

In addition, synthetic biology and native neuronal components, such as supported lipid bilayers, offer promising routes toward cell-like interfaces that mimic not only the softness of neural tissue but also its molecular composition. However, forming stable supported lipid bilayers with native neuron-derived membranes, preserving functional proteins, and integrating them efficiently with neuronal cells and electronic devices remain challenging to achieve.

Another major challenge in achieving minimally invasive living neuroelectronics is cell- or compartment-specific control over polymer formation within neural tissue. In this regard, targeted *in situ* polymerization and GTCA represent particularly promising directions. By localizing enzymes and initiators, or anchoring motifs, to specific cell types or neuronal compartments, synthetic biology could enable conductive polymer formation at specific membranes, axons, dendrites, or synaptic regions. Here, it is needed to reduce dependence on exogenous hydrogen peroxide, improve enzyme activity under physiological conditions, achieve controlled monomer diffusion, and validate long-term effects on neuronal viability, excitability, and network function. Many promising *in situ* polymerization strategies are currently demonstrated *in vitro* or in small model organisms, whereas long-term function in complex mammalian neural tissue remains insufficiently understood. Future work should therefore combine materials development with long-term electrophysiology, histological analysis, polymer degradation studies, and behavioral assessment. In parallel, scalable fabrication methods will be necessary to preserve micro- and nanoscale precision while enabling reproducible device production.

Overall, living neuroelectronics will be achieved through the convergence of organic electronics, polymer chemistry, microfabrication, neuroscience, and synthetic biology. The most promising future systems will not simply contact neural tissue but will become structurally and functionally integrated with it. By combining biomimetic topographies, soft conductive materials, targeted *in situ* polymerization, and GTCA, neurohybrid interfaces may evolve from implanted devices into adaptive living systems capable of long-term communication with the nervous system.

## Author contributions

F. S. and V. C. developed the initial idea and the manuscript outline. All authors performed the literature review and contributed to the writing of the manuscript.

## Conflicts of interest

There are no conflicts to declare.

## Data Availability

No primary research results, software or code have been included as part of this Focus article. The microscopy images of synapses are included for illustrative purposes, and the underlying data are available from the corresponding author upon request.

## References

[cit1] Agnati L. F., Guidolin D., Cervetto C., Maura G., Marcoli M. (2023). Life.

[cit2] Azevedo F. A. C., Carvalho L. R. B., Grinberg L. T., Farfel J. M., Ferretti R. E. L., Leite R. E. P., Filho W. J., Lent R., Herculano-Houzel S. (2009). J. Comp. Neurol..

[cit3] Batool S., Raza H., Zaidi J., Riaz S., Hasan S., Syed N. I. (2019). J. Neurophysiol..

[cit4] Liu X., Hua F., Yang D., Lin Y., Zhang L., Ying J., Sheng H., Wang X. (2022). J. Transl. Med..

[cit5] Krueger-Burg D. (2025). Trends Neurosci..

[cit6] Südhof T. C., Malenka R. C. (2008). Neuron.

[cit7] Zhang A., Zhao S., Tyson J., Deisseroth K., Bao Z. (2024). Nat. Synth..

[cit8] Zhang A., Loh K. Y., Kadur C. S., Michalek L., Dou J., Ramakrishnan C., Bao Z., Deisseroth K. (2023). Sci. Adv..

[cit9] Jastrzebska-Perfect P., Chowdhury S., Spyropoulos G. D., Zhao Z., Cea C., Gelinas J. N., Khodagholy D. (2020). Adv. Funct. Mater..

[cit10] Vassanelli S., Mahmud M. (2016). Front. Neurosci..

[cit11] Bruno U., Mariano A., Rana D., Gemmeke T., Musall S., Santoro F. (2023). Neuromorph. Comput. Eng..

[cit12] Kandel E. R. , Principles of Neural Science, McGraw Hill Professional, 5th edn, 2013

[cit13] Südhof T. C. (2021). J. Cell Biol..

[cit14] Cano-Astorga N., DeFelipe J., Alonso-Nanclares L. (2021). Cereb. Cortex.

[cit15] Samojedny S., Czechowska E., Pańczyszyn-Trzewik P., Sowa-Kućma M. (2022). Int. J. Mol. Sci..

[cit16] Choii G., Ko J. (2015). Exp. Mol. Med..

[cit17] Sheng M., Kim E. (2011). Cold Spring Harbor Perspect. Biol..

[cit18] Leterrier C. (2018). J. Neurosci..

[cit19] Pereda A. E. (2014). Nat. Rev. Neurosci..

[cit20] Bennett M. V. L., Zukin R. S. (2004). Neuron.

[cit21] Chang M., Krüssel S., Parajuli L. K., Kim J., Lee D., Merodio A., Kwon J., Okabe S., Kwon H.-B. (2025). Science.

[cit22] Eroglu C., Barres B. A. (2010). Nature.

[cit23] Demmings M. D., da Silva Chagas L., Traetta M. E., Rodrigues R. S., Acutain M. F., Barykin E., Datusalia A. K., German-Castelan L., Mattera V. S., Mazengenya P., Skoug C., Umemori H. (2025). J. Neurochem..

[cit24] van Oostrum M., Schuman E. M. (2025). Nat. Rev. Neurosci..

[cit25] Gomez A. M., Traunmüller L., Scheiffele P. (2021). Nat. Rev. Neurosci..

[cit26] Bemben M. A., Shipman S. L., Nicoll R. A., Roche K. W. (2015). Trends Neurosci..

[cit27] Chen J. L., Villa K. L., Cha J. W., So P. T. C., Kubota Y., Nedivi E. (2012). Neuron.

[cit28] Chiu C. Q., Lur G., Morse T. M., Carnevale N. T., Ellis-Davies G. C. R., Higley M. J. (2013). Science.

[cit29] Torres V. I., Vallejo D., Inestrosa N. C. (2017). Neural Plast..

[cit30] Nakahata Y., Yasuda R. (2018). Front. Synaptic Neurosci..

[cit31] Bingham D., Jakobs C. E., Wernert F., Boroni-Rueda F., Jullien N., Schentarra E.-M., Friedl K., Da Costa Moura J., Van Bommel D. M., Caillol G., Ogawa Y., Papandréou M.-J., Leterrier C. (2023). J. Cell Biol..

[cit32] de León-López C. A. M., Carretero-Rey M., Khan Z. U. (2025). Cell. Mol. Neurobiol..

[cit33] Turrigiano G. G., Nelson S. B. (2004). Nat. Rev. Neurosci..

[cit34] Südhof T. C. (2018). Neuron.

[cit35] Cerina M., Piastra M. C., Frega M. (2023). Prog. Biomed. Eng..

[cit36] Ghovanloo M.-R., Dib-Hajj S. D., Waxman S. G. (2025). Mol. Pharmacol..

[cit37] Yi D., Yao Y., Wang Y., Chen L. (2022). J. Micro Nano-Manuf..

[cit38] Kim R., Joo S., Jung H., Hong N., Nam Y. (2014). Biomed. Eng. Lett..

[cit39] Woeppel K. M., Cui X. T. (2021). Adv. Healthcare Mater..

[cit40] Aktas B., Ozgun A., Kilickap B. D., Garipcan B. (2024). J. Biomed. Mater. Res., Part B.

[cit41] Fattahi P., Yang G., Kim G., Abidian M. R. (2014). Adv. Mater..

[cit42] Kozai T. D. Y., Jaquins-Gerstl A. S., Vazquez A. L., Michael A. C., Cui X. T. (2015). ACS Chem. Neurosci..

[cit43] Gkoupidenis P., Zhang Y., Kleemann H., Ling H., Santoro F., Fabiano S., Salleo A., van de Burgt Y. (2024). Nat. Rev. Mater..

[cit44] Kaur G., Adhikari R., Cass P., Bown M., Gunatillake P. (2015). RSC Adv..

[cit45] Gao D., Van Der Pol T. P. A., Musumeci C., Tu D., Fabiano S. (2025). Annu. Rev. Chem. Biomol. Eng..

[cit46] Go G.-T., Lee Y., Seo D.-G., Lee T.-W. (2022). Adv. Mater..

[cit47] Namsheer K., Rout C. S. (2021). RSC Adv..

[cit48] Jones P. D., Moskalyuk A., Barthold C., Gutöhrlein K., Heusel G., Schröppel B., Samba R., Giugliano M. (2020). Front. Neurosci..

[cit49] Aqrawe Z., Wright B., Patel N., Vyas Y., Malmstrom J., Montgomery J. M., Williams D., Travas-Sejdic J., Svirskis D. (2019). Sens. Actuators, B.

[cit50] Muguet I., Maziz A., Mathieu F., Mazenq L., Larrieu G. (2023). Adv. Mater..

[cit51] Ludwig K. A., Langhals N. B., Joseph M. D., Richardson-Burns S. M., Hendricks J. L., Kipke D. R. (2011). J. Neural Eng..

[cit52] Ganji M., Elthakeb A. T., Tanaka A., Gilja V., Halgren E., Dayeh S. A. (2017). Adv. Funct. Mater..

[cit53] Khodagholy D., Gelinas J. N., Thesen T., Doyle W., Devinsky O., Malliaras G. G., Buzsáki G. (2015). Nat. Neurosci..

[cit54] Li J., Mo D., Hu J., Wang S., Gong J., Huang Y., Li Z., Yuan Z., Xu M. (2025). Microsyst. Nanoeng..

[cit55] Dijk G., Pas J., Markovic K., Scancar J., O’Connor R. P. (2023). APL Bioeng..

[cit56] Rivnay J., Owens R. M., Malliaras G. G. (2014). Chem. Mater..

[cit57] Beaumont C., Turgeon J., Idir M., Neusser D., Lapointe R., Caron S., Dupont W., D’Astous D., Shamsuddin S., Hamza S., Landry É., Ludwigs S., Leclerc M. (2021). Macromolecules.

[cit58] Xiao Y., Cui X., Martin D. C. (2004). J. Electroanal. Chem..

[cit59] Gu M., Travaglini L., Hopkins J., Ta D., Lauto A., Wagner P., Wagner K., Zeglio E., Jephcott L., Officer D. L., Mawad D. (2022). Synth. Met..

[cit60] Mantione D., Del Agua I., Sanchez-Sanchez A., Mecerreyes D. (2017). Polymers.

[cit61] Fenoy G. E., Azzaroni O., Knoll W., Marmisollé W. A. (2021). Chemosensors.

[cit62] Bhagwat N., Kiick K. L., Martin D. C. (2014). J. Mater. Res..

[cit63] Mantione D., Marquez A. V., Cruciani F., Brochon C., Cloutet E., Hadziioannou G. (2019). ACS Macro Lett..

[cit64] Daugaard A. E., Hvilsted S., Hansen T. S., Larsen N. B. (2008). Macromolecules.

[cit65] Povlich L. K., Cho J. C., Leach M. K., Corey J. M., Kim J., Martin D. C. (2013). Biochim. Biophys. Acta, Gen. Subj..

[cit66] Bu H.-B., Götz G., Reinold E., Vogt A., Schmid S., Segura J. L., Blanco R., Gómez R., Bäuerle P. (2011). Tetrahedron.

[cit67] Hackett A. J., Malmström J., Travas-Sejdic J. (2017). Prog. Polym. Sci..

[cit68] Kousseff C. J., Taifakou F. E., Neal W. G., Palma M., Nielsen C. B. (2022). J. Polym. Sci..

[cit69] Salinas G., Villarroel Marquez A., Idir M., Shinde S., Frontana-Uribe B. A., Raoux M., Lang J., Cloutet E., Kuhn A. (2020). ChemElectroChem.

[cit70] Matino L., Mariano A., Ausilio C., Garg R., Cohen-Karni T., Santoro F. (2022). Nano Lett..

[cit71] Ojovan S. M., Rabieh N., Shmoel N., Erez H., Maydan E., Cohen A., Spira M. E. (2015). Sci. Rep..

[cit72] Ruggiero A., Criscuolo V., Grasselli S., Bruno U., Ausilio C., Bovio C. L., Bettucci O., Santoro F. (2022). Chem. Commun..

[cit73] Buzio M., Gini M., Schneider T. C., Stajkovic N., Ingebrandt S., De Laporte L., Offenhäusser A., Criscuolo V., Santoro F. (2026). npj Flexible Electron..

[cit74] Mariano A., Lubrano C., Bruno U., Ausilio C., Dinger N. B., Santoro F. (2022). Chem. Rev..

[cit75] Lee M. Y., Lee E. S., Ko N. Y., Kim H. J., Kim D.-H., Cha G. D., Koo J. H. (2025). npj Biosens..

[cit76] Choi J. S., Lee H. J., Rajaraman S., Kim D.-H. (2021). Biosens. Bioelectron..

[cit77] Pennacchio F. A., Garma L. D., Matino L., Santoro F. (2018). J. Mater. Chem. B.

[cit78] Yang X., Zhou T., Zwang T. J., Hong G., Zhao Y., Viveros R. D., Fu T.-M., Gao T., Lieber C. M. (2019). Nat. Mater..

[cit79] Mariano A., Bovio C. L., Criscuolo V., Santoro F. (2022). Nanotechnology.

[cit80] Leclech C., Villard C. (2020). Front. Bioeng. Biotechnol..

[cit81] Brunetti V., Maiorano G., Rizzello L., Sorce B., Sabella S., Cingolani R., Pompa P. P. (2010). Proc. Natl. Acad. Sci. U. S. A..

[cit82] Onesto V., Cancedda L., Coluccio M. L., Nanni M., Pesce M., Malara N., Cesarelli M., Di Fabrizio E., Amato F., Gentile F. (2017). Sci. Rep..

[cit83] Fan S., Qi L., Li J., Pan D., Zhang Y., Li R., Zhang C., Wu D., Lau P., Hu Y., Bi G., Ding W., Chu J. (2021). Adv. Healthcare Mater..

[cit84] Slavík J., Čmiel V., Hubálek J., Yang Y., Ren T.-L. (2021). Appl. Sci..

[cit85] Tonazzini I., Masciullo C., Savi E., Sonato A., Romanato F., Cecchini M. (2020). Sci. Rep..

[cit86] Spira M. E., Hai A. (2013). Nat. Nanotechnol..

[cit87] Hai A., Dormann A., Shappir J., Yitzchaik S., Bartic C., Borghs G., Langedijk J. P. M., Spira M. E. (2009). J. R. Soc., Interface.

[cit88] Dipalo M., McGuire A. F., Lou H.-Y., Caprettini V., Melle G., Bruno G., Lubrano C., Matino L., Li X., De Angelis F., Cui B., Santoro F. (2018). Nano Lett..

[cit89] Aalipour A., Xu A. M., Leal-Ortiz S., Garner C. C., Melosh N. A. (2014). Langmuir.

[cit90] Teixeira H., Dias C., Aguiar P., Ventura J. (2021). Adv. Mater. Technol..

[cit91] Liu Y., Li X., Chen J., Yuan C. (2020). Front. Chem..

[cit92] Cho Y. H., Park Y.-G., Kim S., Park J.-U. (2021). Adv. Mater..

[cit93] Latte Bovio C., Matamoros E., Mollo V., Mariano A., Criscuolo V., Santoro F. (2026). Adv. Sci..

[cit94] Radotić V., Bedalov A., Drviš P., Braeken D., Kovačić D. (2019). J. Neural Eng..

[cit95] Robinson J. T., Jorgolli M., Shalek A. K., Yoon M.-H., Gertner R. S., Park H. (2012). Nat. Nanotechnol..

[cit96] Hanson J. N., Motala M. J., Heien M. L., Gillette M., Sweedler J., Nuzzo R. G. (2009). Lab Chip.

[cit97] Shukla S., Schwartz J. L., Walsh C., Wong W. M., Patel V., Hsieh Y.-P., Onwuasoanya C., Chen S., Offenhäusser A., Cauwenberghs G., Santoro F., Muotri A. R., Yeo G. W., Chalasani S. H., Jahed Z. (2024). Microsyst. Nanoeng..

[cit98] Hai A., Shappir J., Spira M. E. (2010). Nat. Methods.

[cit99] Milos F., Belu A., Mayer D., Maybeck V., Offenhäusser A. (2021). Adv. Biol..

[cit100] Lunghi A., Mariano A., Bianchi M., Dinger N. B., Murgia M., Rondanina E., Toma A., Greco P., Di Lauro M., Santoro F., Fadiga L., Biscarini F. (2022). Adv. Mater. Interfaces.

[cit101] Abu Shihada J., Jung M., Decke S., Koschinski L., Musall S., Rincón Montes V., Offenhäusser A. (2024). Adv. Sci..

[cit102] Yoon D., Nam Y. (2025). Adv. Funct. Mater..

[cit103] Yang X., Tsai C.-T., Yang Y., Zhang W., You H., Forró C., Paşca S. P., Cui B. (2026). Nat. Rev. Bioeng..

[cit104] Mattiassi S., Conner A. A., Feng F., Goh E. L. K., Yim E. K. F. (2023). Cells.

[cit105] Xie X., Xu A. M., Leal-Ortiz S., Cao Y., Garner C. C., Melosh N. A. (2013). ACS Nano.

[cit106] Brigo L., Urciuolo A., Giulitti S., Della Giustina G., Tromayer M., Liska R., Elvassore N., Brusatin G. (2017). Acta Biomater..

[cit107] Heo D. N., Lee S.-J., Timsina R., Qiu X., Castro N. J., Zhang L. G. (2019). Mater. Sci. Eng., C.

[cit108] Bierman-Duquette R. D., Safarians G., Huang J., Rajput B., Chen J. Y., Wang Z. Z., Seidlits S. K. (2022). Adv. Healthcare Mater..

[cit109] Sunwoo S.-H., Ha K.-H., Lee S., Lu N., Kim D.-H. (2021). Annu. Rev. Chem. Biomol. Eng..

[cit110] Wang S., Sun C., Guan S., Li W., Xu J., Ge D., Zhuang M., Liu T., Ma X. (2017). J. Mater. Chem. B.

[cit111] Feron K., Lim R., Sherwood C., Keynes A., Brichta A., Dastoor P. (2018). Int. J. Mol. Sci..

[cit112] Sanjuan-Alberte P., Vaithilingam J., Moore J. C., Wildman R. D., Tuck C. J., Alexander M. R., Hague R. J. M., Rawson F. J. (2021). Polymers.

[cit113] Di Lullo E., Kriegstein A. R. (2017). Nat. Rev. Neurosci..

[cit114] Ferro M. D., Proctor C. M., Gonzalez A., Jayabal S., Zhao E., Gagnon M., Slézia A., Pas J., Dijk G., Donahue M. J., Williamson A., Raymond J., Malliaras G. G., Giocomo L., Melosh N. A. (2024). AIP Adv..

[cit115] Steins H., Mierzejewski M., Brauns L., Stumpf A., Kohler A., Heusel G., Corna A., Herrmann T., Jones P. D., Zeck G., von Metzen R., Stieglitz T. (2022). Microsyst. Nanoeng..

[cit116] Du M., Guan S., Gao L., Lv S., Yang S., Shi J., Wang J., Li H., Fang Y. (2019). Small.

[cit117] Lunghi A., Bianchi M., Greco P., Viaro R., Di Lauro M., Fadiga L., Biscarini F. (2025). Adv. Mater. Interfaces.

[cit118] Khan W. U., Shen Z., Mugo S. M., Wang H., Zhang Q. (2025). Chem. Soc. Rev..

[cit119] Qu J., Xie K., Chen S., He X., Wang Y., Chamberlin M., Zhao X., Zhu G., Xu C., Shi P. (2024). Sci. Adv..

[cit120] Pitsalidis C., Pappa A.-M., Boys A. J., Fu Y., Moysidou C.-M., van Niekerk D., Saez J., Savva A., Iandolo D., Owens R. M. (2022). Chem. Rev..

[cit121] Donahue M. J., Sanchez-Sanchez A., Inal S., Qu J., Owens R. M., Mecerreyes D., Malliaras G. G., Martin D. C. (2020). Mater. Sci. Eng., R.

[cit122] Skorupa M., Więcławska D., Czerwińska-Główka D., Skonieczna M., Krukiewicz K. (2021). Polymers.

[cit123] Cui X., Lee V. A., Raphael Y., Wiler J. A., Hetke J. F., Anderson D. J., Martin D. C. (2001). J. Biomed. Mater. Res..

[cit124] Milos F., Tullii G., Gobbo F., Lodola F., Galeotti F., Verpelli C., Mayer D., Maybeck V., Offenhäusser A., Antognazza M. R. (2021). ACS Appl. Mater. Interfaces.

[cit125] Mariano A., Fasolino I., Dinger N. B., Latte Bovio C., Bonadies I., Pezzella A., Ambrosio L., Raucci M. G., Santoro F. (2023). Adv. Mater. Interfaces.

[cit126] Baek S., Green R. A., Poole-Warren L. A. (2014). J. Biomed. Mater. Res., Part A.

[cit127] Kim D.-H., Richardson-Burns S. M., Hendricks J. L., Sequera C., Martin D. C. (2007). Adv. Funct. Mater..

[cit128] Gomez N., Schmidt C. E. (2007). J. Biomed. Mater. Res., Part A.

[cit129] Feyen P., Colombo E., Endeman D., Nova M., Laudato L., Martino N., Antognazza M. R., Lanzani G., Benfenati F., Ghezzi D. (2016). Sci. Rep..

[cit130] ElMahmoudy M., Inal S., Charrier A., Uguz I., Malliaras G. G., Sanaur S. (2017). Macromol. Mater. Eng..

[cit131] Xie W., Pakdel E., Liang Y., Kim Y. J., Liu D., Sun L., Wang X. (2019). Biomacromolecules.

[cit132] Paulin J. V., Graeff C. F.
O. (2021). J. Mater. Chem. C.

[cit133] Barra M., Bonadies I., Carfagna C., Cassinese A., Cimino F., Crescenzi O., Criscuolo V., d’Ischia M., Maglione M. G., Manini P., Migliaccio L., Musto A., Napolitano A., Navarra A., Panzella L., Parisi S., Pezzella A., Prontera C. T., Tassini P. (2016). MRS Adv..

[cit134] Eom T., Ozlu B., Ivanová L., Lee S., Lee H., Krajčovič J., Shim B. S. (2024). Biomacromolecules.

[cit135] Mostert A. B. (2021). Polymers.

[cit136] Pezzella A., Barra M., Musto A., Navarra A., Alfe M., Manini P., Parisi S., Cassinese A., Criscuolo V., d’Ischia M. (2015). Mater. Horiz..

[cit137] Bettinger C. J., Bruggeman J. P., Misra A., Borenstein J. T., Langer R. (2009). Biomaterials.

[cit138] Huang W.-C., Hung C.-H., Lin Y.-W., Zheng Y.-C., Lei W.-L., Lu H.-E. (2022). ACS Biomater. Sci. Eng..

[cit139] Agrawal L., Vimal S. K., Barzaghi P., Shiga T., Terenzio M. (2022). Macromol. Biosci..

[cit140] Migliaccio L., Aprano S., Iannuzzi L., Maglione M. G., Tassini P., Minarini C., Manini P., Pezzella A. (2017). Adv. Electron. Mater..

[cit141] Hasan M. L., Kim G. E., Elnaggar M. A., Yang D. H., Joung Y. K. (2023). Appl. Surf. Sci..

[cit142] Ausilio C., Lubrano C., Rana D., Matrone G. M., Bruno U., Santoro F. (2024). Adv. Sci..

[cit143] Lubrano C., Bruno U., Ausilio C., Santoro F. (2022). Adv. Mater..

[cit144] Ulmefors H., Nissa J., Pace H., Wahlsten O., Gunnarsson A., Simon D. T., Berggren M., Höök F. (2021). Langmuir.

[cit145] Maraj J. J., Schafer E. A., Mansour M. M., Hussein E. A., Berryman J., Klavon E., Rivnay J., Sarles S. A. (2025). Adv. Electron. Mater..

[cit146] Ge G., Wang W., Wang Q., Wang M., Wang T., Yu L., Zhang X., Zhu C., Xu Y., Yang H., Bai J., Pan G., Geng D. (2024). Adv. Funct. Mater..

[cit147] Pansani T. N., Phan T. H., Lei Q., Kondyurin A., Kalionis B., Chrzanowski W. (2021). Nanomaterials.

[cit148] Staufer O., De Lora J. A., Bailoni E., Bazrafshan A., Benk A. S., Jahnke K., Manzer Z. A., Otrin L., Díez Pérez T., Sharon J., Steinkühler J., Adamala K. P., Jacobson B., Dogterom M., Göpfrich K., Stefanovic D., Atlas S. R., Grunze M., Lakin M. R., Shreve A. P., Spatz J. P., López G. P. (2021). eLife.

[cit149] Gu A. A., Uçar M. C., Tran P., Prindle A., Kamat N. P., Steinkühler J. (2025). Nat. Commun..

[cit150] Lira R. B., Steinkühler J., Knorr R. L., Dimova R., Riske K. A. (2016). Sci. Rep..

[cit151] Gao Z., Schäfer S., Stockmann R., Hoffmann B., Merkel R., Offenhäusser A., Ingebrandt S. (2026). Biosens. Bioelectron..

[cit152] Max K., Sames L., Ye S., Steinkühler J., Corradi F. (2025). Neuromorph. Comput. Eng..

[cit153] Petty A. J. I., Keate R. L., Jiang B., Ameer G. A., Rivnay J. (2020). Chem. Mater..

[cit154] Tommasini G., De Simone M., Blasio M., Zenna C., Tino A., Stavrinidou E., Santillo S., Tortiglione C. (2025). Adv. Mater. Interfaces.

[cit155] Niu J., Lunn D. J., Pusuluri A., Yoo J. I., O’Malley M. A., Mitragotri S., Soh H. T., Hawker C. J. (2017). Nat. Chem..

[cit156] Peramo A., Urbanchek M. G., Spanninga S. A., Povlich L. K., Cederna P., Martin D. C. (2008). Tissue Eng., Part A.

[cit157] Strakosas X., Biesmans H., Abrahamsson T., Hellman K., Ejneby M. S., Donahue M. J., Ekström P., Ek F., Savvakis M., Hjort M., Bliman D., Linares M., Lindholm C., Stavrinidou E., Gerasimov J. Y., Simon D. T., Olsson R., Berggren M. (2023). Science.

[cit158] Sessler C. D., Zhou Y., Wang W., Hartley N. D., Fu Z., Graykowski D., Sheng M., Wang X., Liu J. (2022). Sci. Adv..

[cit159] Walton A. , Biopolymers, Academic Press, 2012

[cit160] Richardson-Burns S. M., Hendricks J. L., Martin D. C. (2007). J. Neural Eng..

[cit161] Ouyang L., Shaw C. L., Kuo C., Griffin A. L., Martin D. C. (2014). J. Neural Eng..

[cit162] Stavrinidou E., Gabrielsson R., Nilsson K. P. R., Singh S. K., Franco-Gonzalez J. F., Volkov A. V., Jonsson M. P., Grimoldi A., Elgland M., Zozoulenko I. V., Simon D. T., Berggren M. (2017). Proc. Natl. Acad. Sci. U. S. A..

[cit163] Wojtovich A. P., Foster T. H. (2014). Redox Biol..

[cit164] Tommasini G., Dufil G., Fardella F., Strakosas X., Fergola E., Abrahamsson T., Bliman D., Olsson R., Berggren M., Tino A., Stavrinidou E., Tortiglione C. (2022). Bioact. Mater..

[cit165] Biesmans H., Farinotti A. B., Abrahamsson T., Arja K., Lindholm C., Strakosas X., Gerasimov J. Y., Simon D. T., Svensson C. I., Musumeci C., Berggren M. (2024). Sci. Adv..

[cit166] Dufil G., Parker D., Gerasimov J. Y., Nguyen T.-Q., Berggren M., Stavrinidou E. (2020). J. Mater. Chem. B.

[cit167] Tommasini G., Simone M. D., Santillo S., Dufil G., Iencharelli M., Mantione D., Stavrinidou E., Tino A., Tortiglione C. (2023). Sci. Adv..

[cit168] Liu J., Kim Y. S., Richardson C. E., Tom A., Ramakrishnan C., Birey F., Katsumata T., Chen S., Wang C., Wang X., Joubert L.-M., Jiang Y., Wang H., Fenno L. E., Tok J. B.-H., Paşca S. P., Shen K., Bao Z., Deisseroth K. (2020). Science.

[cit169] Lim K. H., Huang H., Pralle A., Park S. (2013). Biotechnol. Bioeng..

[cit170] Shu X., Lev-Ram V., Deerinck T. J., Qi Y., Ramko E. B., Davidson M. W., Jin Y., Ellisman M. H., Tsien R. Y. (2011). PLoS Biol..

[cit171] Zhang A., Jiang Y., Loh K. Y., Bao Z., Deisseroth K. (2023). Nat. Rev. Bioeng..

[cit172] Lam S. S., Martell J. D., Kamer K. J., Deerinck T. J., Ellisman M. H., Mootha V. K., Ting A. Y. (2015). Nat. Methods.

[cit173] Hung V., Udeshi N. D., Lam S. S., Loh K. H., Cox K. J., Pedram K., Carr S. A., Ting A. Y. (2016). Nat. Protoc..

[cit174] Lee S.-Y., Roh H., Gonzalez-Perez D., Mackey M. R., Hoces D., McLaughlin C. N., Lin C., Adams S. R., Nguyen K., Kim K.-Y., Luginbuhl D. J., Luo L., Udeshi N. D., Carr S. A., Hernández-López R. A., Ellisman M. H., Alcalde M., Ting A. Y. (2025). Nat. Chem. Biol..

